# Reconciling Chain Orientation in Polymer-Grafted Nanoparticles between Coarse-Grained Models and Resonant Soft X-ray Scattering

**DOI:** 10.1021/acsnano.4c18022

**Published:** 2025-04-17

**Authors:** Subhrangsu Mukherjee, Nicholas T. Liesen, Scott T. Milner, Lisa M. Hall, Dean M. DeLongchamp

**Affiliations:** † Materials Science and Engineering Division, Materials Measurement Laboratory, National Institute of Standards and Technology, Gaithersburg, Maryland 20899, United States; ‡ Physical and Life Sciences Directorate, Lawrence Livermore National Laboratory, Livermore, California 94550, United States; § Department of Chemical Engineering, The Pennsylvania State University, University Park, Pennsylvania 16802, United States; ∥ Department of Chemical and Biomolecular Engineering, The Ohio State University, Columbus, Ohio 43221, United States

**Keywords:** nanoparticle, polymer, polymer-grafted nanoparticles, orientation, coarse-grained, X-ray scattering, polarized soft X-ray scattering

## Abstract

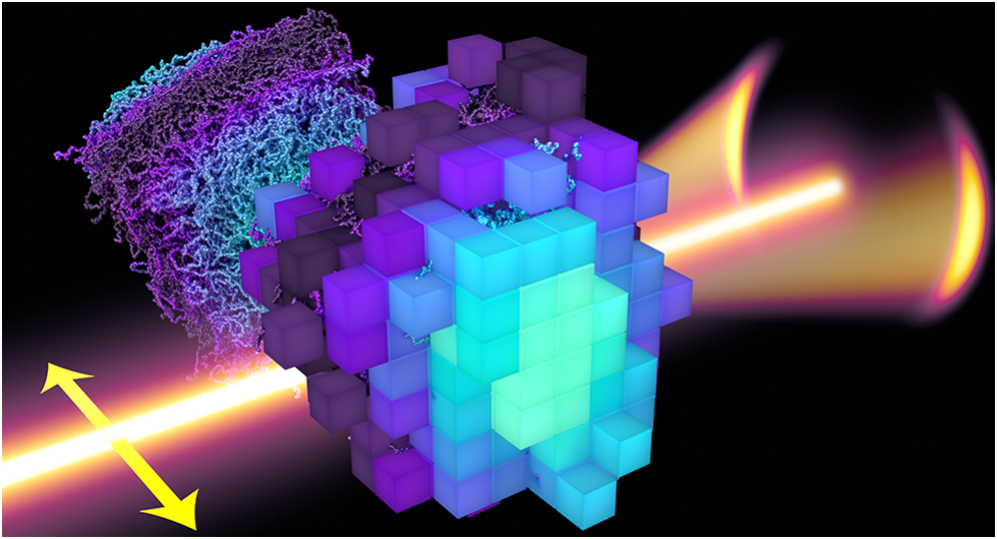

Polymer chain stretching enables the plastic and elastic properties that make polymers unique and valuable engineering materials. Despite its importance, polymer chain orientation in amorphous regions remains very challenging to measure by conventional techniques because it is an intrinsically molecule-scale phenomenon lacking long-range order that is frequently heterogeneous across length scales of ≈ (1 to 100) nm. Polarized resonant soft X-ray scattering (P-RSoXS) is an emerging technique that has recently achieved the measurement of amorphous chain orientation with ≈2 nm spatial resolution. The advent of this measurement capability invites comparisons with computational results for which spatial variations in chain orientation are readily accessible, providing a powerful approach to computation validation. Here we forward simulate P-RSoXS patterns for polystyrene grafted gold nanoparticles from real-space representations incorporating spatial polymer backbone orientation heterogeneity directly extracted from coarse-grained modeling results. Agreement between the computation and P-RSoXS experiment is found to depend greatly on assumptions of phenyl ring conformation relative to the polymer chain backbone, because the orientation sensitivity of P-RSoXS relies on a bond-level transition dipole moment of the phenyl ring of polystyrene to report backbone orientation. By incorporating a statistical description of phenyl ring orientation based on atomistic calculations, we report excellent agreement between P-RSoXS data and forward-simulated patterns with no fitting variables.

Polymer chain stretching is a pervasive concept that has been understood since the earliest days of polymer science^1^ to explain the origins of plastic and elastic behavior that make polymers valuable and unique among materials. The coiled conformation of polymer backbones in solution or melt exhibits an entropic elastic constant that provides a restoring force upon chain stretching. The entropic driving force for return from a stretched to a conformationally random chain underlies Rouse dynamics.^2,3^ Combined with considerations of entanglement^4,5^ and excluded volume,^6^ the concept of chain stretching underlies our modern understanding of polymer mechanics. Broad categories of commercially important polymeric materials are based on the physics of chain stretching. Soft segment chain stretching drives the elasticity of thermoplastic elastomers,^7^ and chain stretching among amorphous stress transmitters in interlamellar amorphous polymer provides the durability and ductility of commodity semicrystalline polyolefins.^8^ Chain stretching is also central to recent explorations of polymer chain behavior in confinement. In grafted polymer brushes, chain stretching physics are a principal contributor to experimental observations of graft surface forces,^9^ and they are a key element of theoretical descriptions of such brushes.^10^ The same concepts apply to systems with nanoscale confinement such as thin polymer films,^11^ star polymers,^12^ polymer-grafted nanoparticles (PGNs),^13,14^ and bottlebrush polymers,^15,16^ all of which exhibit emergent properties due to polymer chain stretching.

Despite the importance of polymer chain stretching, spatially resolved measurements of chain orientation are quite rare.^17^ Chain stretching is an intrinsically molecule-scale phenomenon requiring a high degree of spatial resolution to capture its heterogeneity. Most early theoretical descriptions of chain stretching were informed by polymer chain behavior in dilute solution,^4,5^ in which stretching was measured indirectly with neutron scattering measurements of compositional heterogeneity, perceiving an averaged reciprocal space density silhouette of the coil and deducing chain stretching behavior from its departure from the conformational characteristics of random coils. Macroscopic chain orientation can be measured optically or spectroscopically with dichroism or birefringence, but these approaches are not spatially resolved and will mix signals from different nanoscale regions such as crystalline vs noncrystalline, hard segment vs soft segment, or confined vs unconfined regions. Fiber scattering approaches only capture orientation distributions of crystals from which the orientation of amorphous chains may be indirectly deduced. Coarse-grained modeling of polymer chains delivers explicit chain stretching information,^18^ but that information is typically reconciled with experiment via measurements of structure or dynamics that are based on composition/density fluctuations, from which chain stretching is, again, indirectly deduced.

Polarized resonant soft X-ray scattering (P-RSoXS) is a newly developed technique with the potential to measure chain orientation in polymers with nanometer-scale resolution. P-RSoXS is a reciprocal space measurement method that shares principles with small-angle neutron scattering (SANS) and small-angle X-ray scattering (SAXS). Like those methods, it provides a reciprocal space description of material heterogeneity averaged across the measured ensemble; the phase of scattered radiation is lost, so direct reconstruction of real space structure is not possible and the scattering result must be analyzed via models. Where P-RSoXS differs dramatically from SAXS and SANS is in its contrast mechanisms: the technique derives contrast from intrinsic differences in the soft X-ray index of refraction across low atomic number elemental edges such as the Carbon, Nitrogen, and Oxygen K-edges. Resonant soft X-ray excitations have transition dipole moments, and the optical properties that lead to scattering contrast depend on the orientations of those transition dipoles and their interactions with the imposed (typically linear) electric field vector. P-RSoXS therefore offers scattering contrast based on bond orientation, a capability that is unique among small-angle scattering methods.

The application of P-RSoXS to measure polymer chain stretching requires an examination of polymer chain chemistry, and considerations may be different for each polymer because the resonant soft X-ray excitations and their transition dipole moments are bond-specific. Some bonds may describe the polymer backbone chain axis, and some may not. To measure polymer chain stretching with P-RSoXS therefore has some prerequisites. First, the polymer primary chemical structure must have energetically separable bond transition dipole moments that report the chain axis orientation. These might include bonds that are directly part of the chain axis, or bonds with or within side groups that have a predictable relationship to the chain axis. The most common chain axis bonds, carbon–carbon single bonds, have 1s → σ* (C–C) resonances, and although it is possible in principle to separate 1s → σ* (C–C) resonances with different origins, they are the least likely to be practically separable from side group contributions. Further, 1s → σ* (C–C) resonances occur in a spectral region of significant absorbance, making the P-RSoXS measurement challenging to collect. For polymers with carbon–carbon single bond backbones, therefore, unique resonances of the side groups may instead be used to measure chain axis orientation. This approach leads to a second prerequisite, which is that developing relationships between individual bond orientation and polymer chain orientation frequently requires an assumption of a specific molecular conformation or conformational ensemble. In P-RSoXS analysis, these assumptions are typically included in the development of descriptions of their anisotropic indices of refraction, specifically uniaxial or biaxial descriptions that assert a specific molecular arrangement (or ensemble thereof) with respect to the optical axes.

We previously developed a framework to quantitatively analyze P-RSoXS data to extract the spatial extent of chain orientation in PGNs composed of gold nanoparticle cores with polystyrene (PS) brushes.^19^ PGNs are a rapidly growing product class with a wide variety of industrial applications in the aerospace, automobile, tire, food packaging and biomedical industries.^20−30^ Because tailoring the grafted polymer chain attachment density and particle geometry provide exquisite control over chain stretching, PGNs have also become a platform with which to explore fundamental polymer physics concepts. Ohno et al.^31,32^ have extended the Daoud-Cotton^12^ model to predict the conditions under which chains are stretched and confined for solvated PGNs. Close enough to the nanoparticle and at high enough graft density, the polymer brush is densely crowded, a scenario referred to as the concentrated polymer brush (CPB) regime. Further from the particle, long enough chains will enter the semidilute polymer brush (SDPB) region where the solvent conditions become relevant (in PGN melt systems, the situation is somewhat different as grafts on other PGNs serve as the only ’solvent’).

Molecular modeling efforts have provided more detailed insights into these regimes. For example, ref (33). uses atomistic simulations of Au-PEO PGNs in water to explore the brush regimes, quantifying local dehydration of the brush; they find scaling consistent with the Daoud-Cotton model, including local PEO densities that decrease as *r*^4/3^ in densely grafted systems. They also report that chain orientation and stretching produces a *N*_graft_^4/5^ scaling in brush height with grafted chain length (*N*_graft_), consistent with the measured behavior of confined chains in the CPB regime (and stronger than the *N*_graft_^3/5^ scaling of a SDPB). In another study of dilute PGNs in a medium, in this case in a polymer matrix, ref (34). employed a coarse-grained Monte Carlo method to characterize the structure and scaling of a polystyrene brush on a silica nanoparticle in free polystyrene. This model shows graft density profiles (vs distance from the NP) with features corresponding to the CPB and SDPB regimes, and estimates scaling relationships for brush thickness with graft density and degree of polymerization.^34^ In more recent work using coarse-grained MD, Midya et al. have proposed a related two-layer model to characterize the behavior of PGN melts.^35^ The model proposes the presence of two distinct regions within the polymer brush of PGNs. The inner region is characterized by a high degree of confinement and is devoid of any other polymer chains from surrounding PGNs. The outer region is less constrained, allowing penetration from other polymer chains.

Further insights into the effects of grafted chain conformation on dynamics were provided by recent systematic core-modified dissipative particle dynamics (CM-DPD) simulations.^36^ These showed that grafted chains relax more slowly than free chains and that the relaxation time of the grafted chains scales inversely with the confinement strength.^36^ In the case of PGNs in solution, the relaxation process is Rouse-like or Zimm-like, whereas in a PGN melt the relaxation processes are always Rouse-like.

These examples represent a larger body of work by multiple groups in which molecular models have shown utility to provide scaling laws for different regimes of conformational behavior, directly measure chain statistics and orientation, and explore the effects of NP surface curvature, graft density, degree of polymerization, and polydispersity index,^37^ among other factors. These models have spanned a range of approaches, from atomistically detailed MD calculations,^33,38^ to coarse grained dynamics,^35,36,39−45^ Monte Carlo,^37^ and hybrid approaches that mix length scales.^34^ We point the interested reader to a number of reviews to more broadly explore progress in modeling, experimental synthesis, and characterization around PGN conformational behavior and structure–property relationships, including refs (46−49). Notably, chain stretching is generally examined indirectly via dynamics measurements and simulations, and thus, direct evidence for chain stretching due to confinement in dry solid PGN films is still lacking. The topic is of paramount importance, as it is closely tied to the system’s mechanical performance. The mechanical properties of these systems depend on entanglement among adjacent neighbors and stretching of the polymer chains due to confinement.

Here, we return to the P-RSoXS measurement of PGNs with an analysis framework that provides direct links between the P-RSoXS measurement of chain stretching and computational results. We start with coarse-grained (CG) simulations tailored to our measured PGN monolayers; time averaged snapshots of the two PGN systems are shown in Figure 1. Using our P-RSoXS modeling framework, the NIST RSoXS Simulation Suite (NRSS),^50^ we are then able to take high-resolution, voxelized real-space representations of chain stretching in CG simulations, and simulate the expected P-RSoXS behavior. Stark discrepancies between the initial simulated pattern and experimentally collected data force us to confront and improve assumptions about PS molecular conformation—specifically the orientation of the phenyl ring relative to the backbone—that we made in our earlier work. Here we improve our soft X-ray uniaxial index of refraction using the results of both CG (monomer-level) and atomistic simulations. We combine this information into a hybrid model containing nanoparticle locations from atomic force microscopy (AFM), experimental spectra from near edge X-ray absorption fine structure (NEXAFS) spectroscopy, chain orientation predictions from CG methods, and conformational predictions from atomistic simulations. This data fusion model predicts the measured P-RSoXS patterns with accuracy and without any fitting parameters, validating the CG description of chain stretching and providing a potentially more accurate and better validated description of chain orientation than our earlier parametric models. This combination of experiment and theory opens up a frontier of chain stretching investigation that leverages computation and the unique contrast modalities of the P-RSoXS experiment.

**Figure 1:**
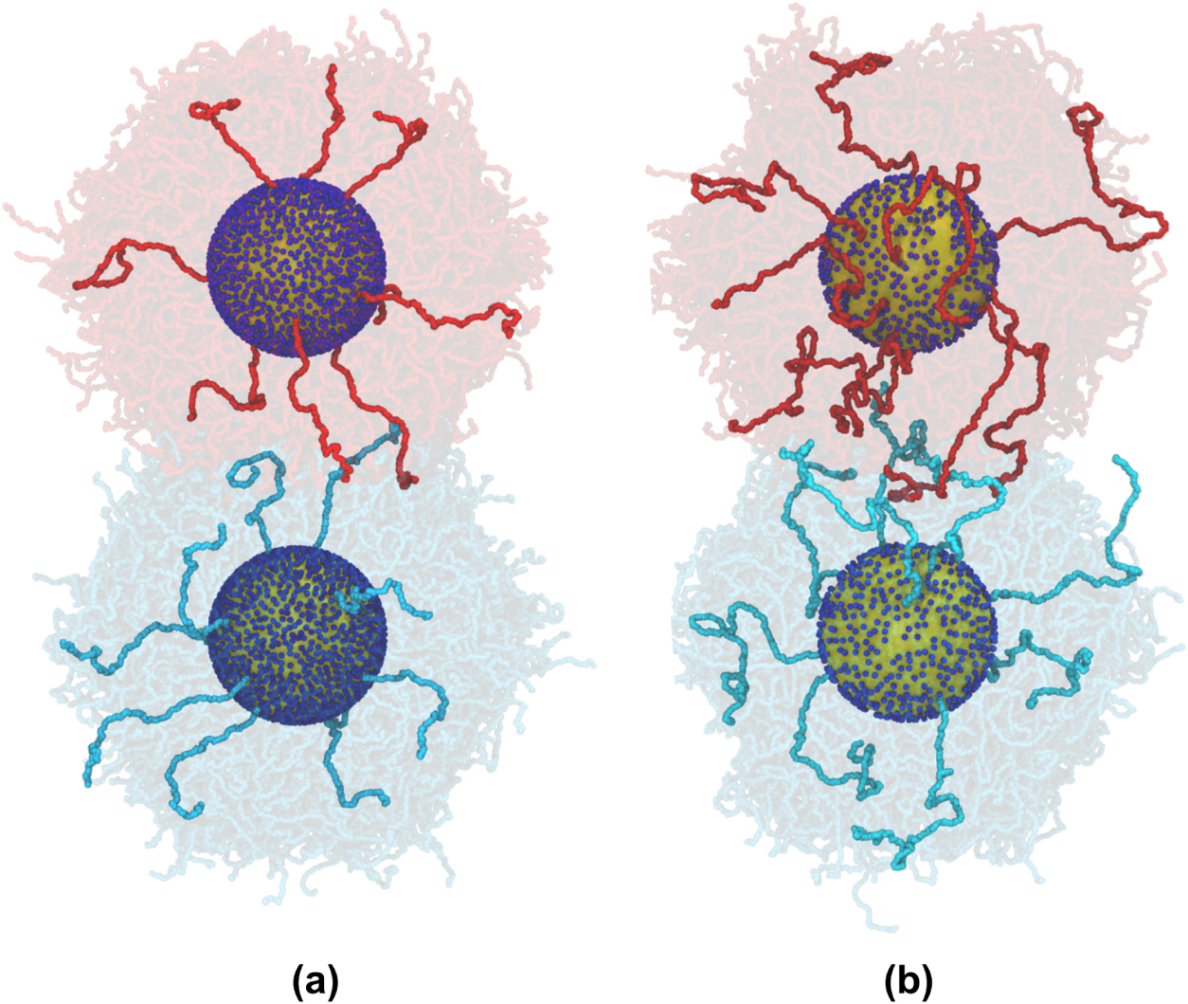
Snapshots of two particle systems simulated using CG simulations: (a) the high graft density (1.2 chains nm^–2^), shorter chain (27 kDa) system (AuPS27), and (b) the low graft density (0.6 chains nm^–2^), longer chain (53 kDa) system (AuPS53). Both systems include a gold particle of radius 10 nm at center. Chains are colored red or blue based on the particle to which they are grafted, and they are wrapped into the image with that particle; a few selected chains are shown solid while the rest are transparent. Chain conformations are averaged over 5 equally spaced frames covering 2500τ of the data collection period described in the text. Dark blue dots are used to visually represent grafting points for the chains. Snapshots were created using Visual Molecular Dynamics (VMD).^51^

## Results and Discussion

A series of coarse-grained molecular dynamics simulations are used as a starting point for fitting-parameter-free prediction of PGN grafted chain orientation. The simulated nanoparticles’ interparticle distances (in the hexagonally packed arrangement considered) are 43.1 ± 0.1, and 43.9 ± 0.2 nm for the AuPS27 and AuPS53 systems, respectively; these interparticle distances are similar due to the roughly equal amounts of polymer in these two systems (i.e., chain length is doubled when surface graft density is halved).

**Figure 2:**
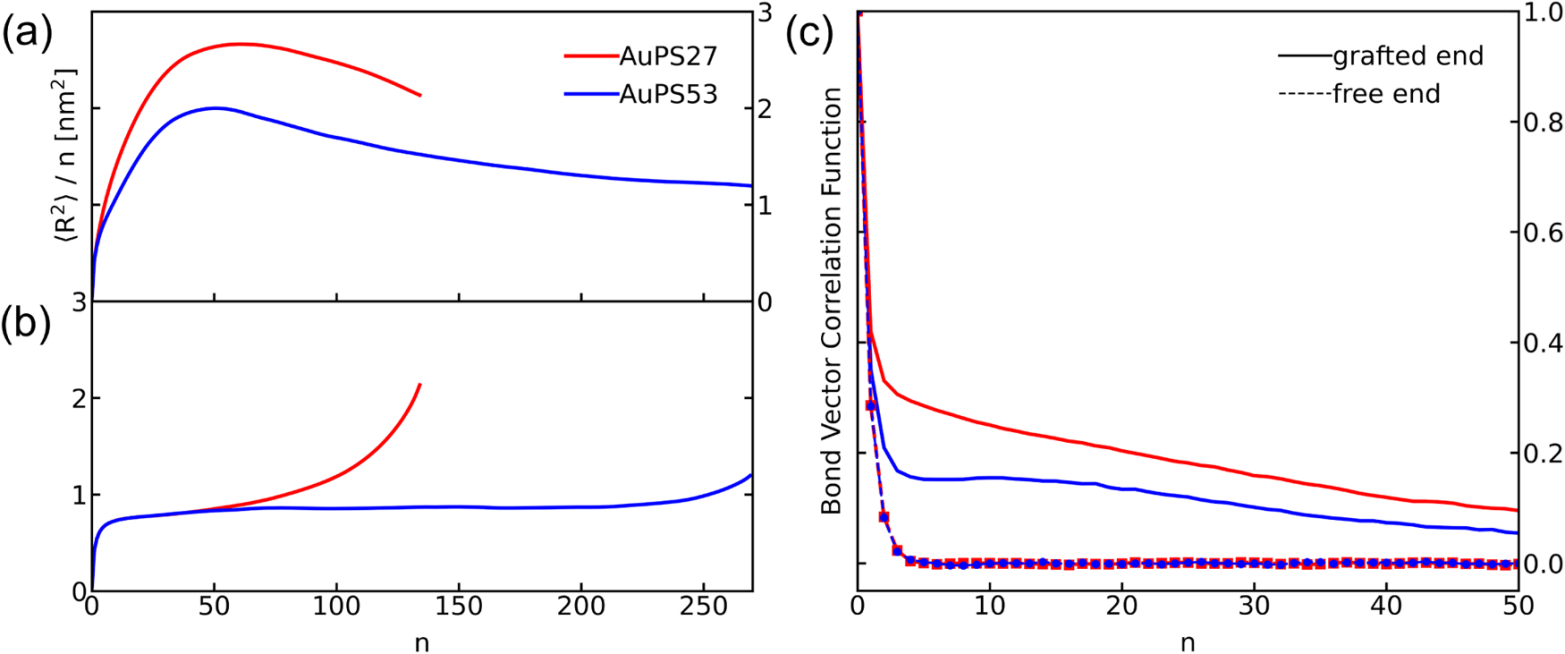
Mean square internal distances from molecular dynamics configurations (mapped to nm^2^ using 0.67 nm/σ) normalized by chemical distance, *n* (in beads, which map to PS as 1.9 PS monomers/bead), for the high AuPS27 (red line) and low AuPS53 (blue line) graft density systems. Chemical distances are measured from the (a) grafted, and (b) free end of the chain. Bond-vector correlation functions are shown in (c) for the two systems, starting from the grafted end (solid lines), and the free end (dashes with symbols).

To supplement orientation fields, we calculate internal squared distances for the grafted chains as described in the Methods section; these pairwise internal distances characterize grafted chain conformations for the AuPS27 (red in Figure 2) and AuPS53 (blue) PGNs. The internal distance profile in Figure 2a is calculated such that we start from the chain end nearest to the nanoparticle’s surface (monomer 1) and consider monomer 1 + *n*, as described in detail within the Methods section, and then starting from the last monomer (*N*) and considering monomer *N* – *n* in Figure 2b (i.e., starting from the free end of the chain).

The differences in grafting density, and the transitions between more concentrated (i.e., highly extended chains packed such that the neighboring brush’s monomers are excluded) and interpenetrated portions of the brush are visible in these internal distance profiles. Specifically, at short length scales (near the surface where the brush is most concentrated), chains are highly extended away from the nanoparticle surface, and internal distances increase rapidly, as shown in Figure 2a. In the higher graft density AuPS27 system, the increase is faster, and the internal distances are larger overall, as we would expect due to the more concentrated brush; these results are consistent with the overall higher degree of alignment with the radial director evident in the orientation profiles we will discuss below. Note that the downturn in the per-monomer end-to-end distance is an expected geometric effect of walking further along a random walk after first walking in a straighter path from the initial point; as shown in Figure 2b, the same quantity measured from the free end of the chain, the chain conformations near the free ends follow nearly random walk statistics. Chain extension from the grafted end is also shown in the bond vector correlation plots in Figure 2c; the solid lines, calculated from the grafted end of the chain, show longer-range decorrelation of bond vectors. This decorrelation is longer-ranged in the higher graft density AuPS27 system versus the lower graft density AuPS53 system, which is similarly consistent with an overall higher degree of grafted chain orientation in this system. The higher chain extension of AuPS27 is also reflected in the overall chain dimensions. Despite the AuPS53 chains being twice the length of the AuPS27 chains, their average end-to-end distance is only 1.6 nm longer (26.8 nm versus 25.2 nm respectively). On a per-monomer basis, this corresponds to a final relative extension of 2.1 nm^2^ versus 1.2 nm^2^ for AuPS27 and AuPS53, respectively in Figure 2.

Considering the distance from the free end of the chain instead in (Figure 2b), the initial random walk regime at high n far from the particle is followed by a rapid increase in normalized extension over the last 50 to 60 monomers near the grafted end of the chain. Similarly, the observed exponential decays of the bond vector correlation functions are identical for the two molecular masses when starting from the free end of the chain (symbols and dashed lines), suggesting more bulk-like behavior.

**Figure 3:**
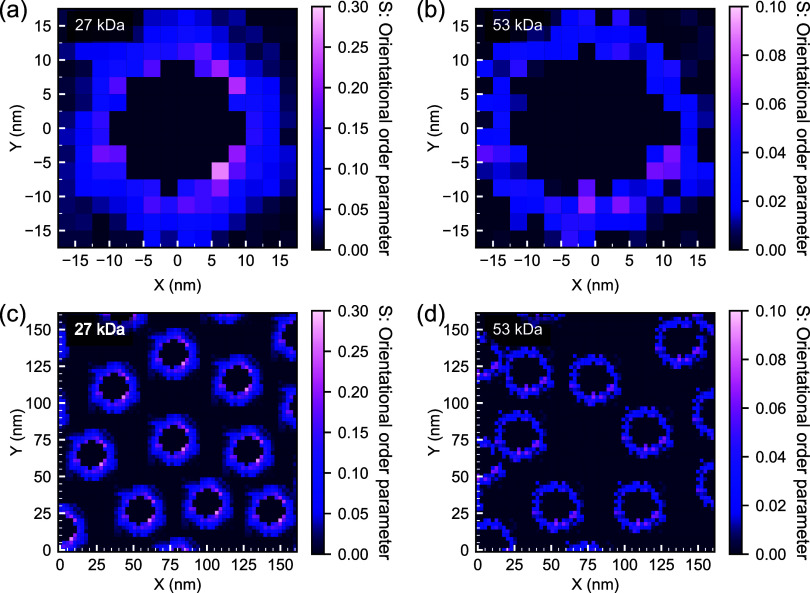
Cross-sectional views of a subsection of the (a) AuPS27 and (b) AuPS53 morphology models showing the stamp placement at locations extracted from AFM imaging. The color bars indicate the orientational order parameter *S*, which may be interpreted in this context as the volume fraction within each voxel of chains perfectly oriented in the radial direction, balanced by a volume fraction of 1 – S isotropic chains. *S* may alternatively be interpreted as describing the extent of radial orientation, with *S* = 1 describing perfect orientation and *S* = 0 describing isotropic chains. Voxels with *S* = 0 in the center of each particle represent the gold core.

The chain conformations extracted from these bead–spring models were then used to generate orientation profiles for the chains for direct embedding into real-space models for P-RSoXS simulation using the NRSS framework. The NRSS is a P-RSoXS-focused member of an emerging family of small-angle scattering (SAS) analysis methods—such as the powerful CREASE method^52^—that exploit the data fusion potential of real-space models incorporating multimodal experimental results and/or simulation results to yield nanoscale structural models that are more information-rich and more unique than conventional SAS analysis can deliver. Our conversion of the CG results into an NRSS model assumes that the most common orientation within a voxel is radial with respect to the nanoparticle center and that the extent of common orientation within each voxel is accurately expressed by *S*, an orientational order parameter that can vary from 0 (isotropic) to 1 (perfectly radially oriented). For each particle, single-particle *S*-fields (or “stamps”) are extracted from the coarse-grained orientation fields (Figure 3a,3b). These stamps can then be repeated at each AFM-derived nanoparticle position, leading to the *S*-field cross sections shown in Figure 3c,d. We observe that *S* decays to ≈zero within 10 nm of the particle surface, and the maximum *S* value is lower compared to the value obtained from the parametric fits to the experimental data in our previous work for both graft densities.^19^ The PS density used in the P-RSoXS simulation was uniform; although it is true that subtle density heterogeneity can affect P-RSoXS processes, the *S*-field is expected to have a significantly more profound effect. The *S*-field describes the strength of orientation of a uniaxial index of refraction oriented in the radial direction. Its representation of the molecular conformation(s) within the voxel, however, and the impact of that representation on simulated P-RSoXS patterns, depends on the assumptions made for soft X-ray index of refraction development, which we will address more completely below.

To simulate P-RSoXS patterns of oriented polymers or molecules, we need an energy-dependent three-dimensional (3D) tensor connecting the structure and degree of orientation of the molecule to the real and imaginary parts of the tensor. The index of refraction in the X-ray regime can be expressed as *n* = 1 – δ + *i*β. The energy dependence of the imaginary part (β) was obtained from NEXAFS spectra of a PS film, and a Kramers–Krönig transformation provides the real part (δ).^53−55^ However, the molecular geometry must be considered to break the real and imaginary parts into a 3D tensor. In our previous work,^19^ we developed a uniaxial description of the complex index of refraction for PS. A “uniaxial” description has one unique (extraordinary) optical axis, with the other two axes of three spatial dimensions sharing the same (ordinary) optical properties. Our complex index of refraction represented the extraordinary direction as the PS backbone direction, the direction of a hypothetical all-*anti*-conformation PS backbone. In this description, taking into account amorphousness, we assumed that the phenyl groups might adopt any fixed rotation about their sp^3^ attachment points to the backbone. An ensemble of differently rotated 1s → π* transition dipoles will therefore describe a plane perpendicular to the sp^3^ attachment bond of the phenyl ring to the backbone, and all rotations about the backbone axis are equally possible. This geometrically idealized axially symmetric uniaxial tensor has an extraordinary index of refraction parallel to the backbone with a higher 1s → π* absorbance (1.5 × that of isotropic PS), whereas the ordinary axes orthogonal to the backbone have a lower 1s → π* absorbance (0.75 × that of isotropic PS). The corresponding δ and β are shown in Figure S1. Henceforth, we shall refer to this geometrically idealized phenyl ring conformation as “axially symmetric”.

**Figure 4:**
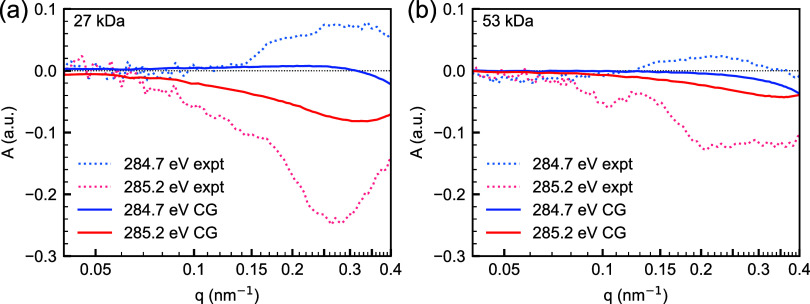
Comparison of experimental (expt) and simulated (CG) *q*-dependence of anisotropy parameter *A*(*q*) at 284.7 and 285.2 eV for the (a) low Mn and high graft (AuPS27), and (b) high Mn and low graft (AuPS53) systems assuming radial chains. Simulations were done on the morphologies obtained from CG simulations shown in Figure 3. The axially symmetric assumption was used to calculate the uniaxial index of refraction. For the definition of *A*(*q*), see the Methods section.

The scattering simulation results are shown in Figure 4. Similar to the experiment, the P-RSoXS simulation exhibits the greatest anisotropy at energies near ≈285 eV, which corresponds to the PS phenyl (1s → π*) NEXAFS transition. To study the orientational aspects of the scattering, we examine the *q*-dependence of the anisotropy, *A*(*q*), which was extracted from simulated patterns. The *A*(*q*) is an abstraction of scattering data that we apply here as it is defined in our previous work;^19^ this scattering-intensity-independent transform of P-RSoXS data is extremely sensitive to anisotropy details of the corona. The simulated *A*(*q*) has a peak position at a slightly lower *q* value compared to the experiment. The major discrepancy is observed in the simulated *A*(*q*) peak magnitude, which is ≈0.25 × that of the experiment.

The sign of *A*(*q*) at the energies of comparison is correct and consistent between the CG computation and the experimental P-RSoXS pattern, confirming a net radial orientation of PS chains. One of the few other measurement techniques to approach nanoscale segmental orientation in grafted systems—nuclear magnetic resonance (NMR)—has found “motional uniaxiality” preferentially tangential to the surface of grafted brushes.^56^ We note that P-RSoXS measures the static ensemble optical properties of the PS rather than its dynamics; the comparison of results between these techniques suggests that the steady-state orientation and the direction of orientational fluctuations may be orthogonal. More comparisons on systems of similar graft density are necessary to further elucidate the relationships.

The discrepancy between simulated and experimental P-RSoXS pattern anisotropy indicates either that the CG computation dramatically under-represents the real extent of chain stretching or that the assumptions made in the P-RSoXS simulation workflow cause the simulated anisotropy to be erroneously low. To better reconcile the simulation and experiment, we examine the latter possibility. The assumption of an axially symmetric conformational ensemble of phenyl rings is the simplifying assumption that has the most profound effect on the magnitude of simulated pattern anisotropy in our P-RSoXS workflow. There are ample reasons to suspect that an axially symmetric description would be incorrect. In isotactic^57^ and syndiotactic^58,59^ polystryrene crystal structures, the phenyl ring is typically found with a marked preference for the phenyl ring normal to be nearly parallel to the polymer chain backbone, though it is true that intramolecular packing considerations may influence this orientation in crystals. There is, in addition, a rich literature addressing phenyl ring orientation in atactic, amorphous polystyrenes which also finds the phenyl ring frequently preferentially oriented.^55,60,61^ We therefore critically examine this assumption and consider alternative index of refraction descriptions.

To investigate how the phenyl rings in polystyrene orient with respect to the backbone direction, atomistic simulations are required. However, these simulations need not be performed on the full system of grafted chains. Rather, we contend that the orientation distribution of phenyl ring normals with respect to the backbone is a rather local property, only weakly perturbed by the relatively modest chain orientation and stretching induced by the grafting. Thus, we can measure the orientation distribution in a more manageable atomistic simulation, of a melt of polystyrene oligomers.

To perform these atomistic simulations, we constructed 64 atactic polystyrene chains of 20 monomers each, initially in all-trans configurations with locally energy-minimized geometries, packed in a loose 8 × 8 × 1 array of parallel chains. The system was then energy minimized, and then slowly resized at 500 K over several nanoseconds to experimental melt density, during which the loosely packed chains have ample opportunity to adopt random-coil configurations. After this, the system was equilibrated for 50 ns at 500 K and fixed pressure. In these simulations the Optimized Potentials for Liquid Simulation (OPLS)^62^ force field was used, with the backbone dihedral potentials adjusted to achieve a good match between experimental persistence lengths (approximated as half the Kuhn length) and the persistence length inferred from the exponential decay of the backbone tangent–tangent correlation function. Finally, the phenyl ring orientation distribution was measured, with the relevant angle defined as the angle between the phenyl ring normal and the vector between next-nearest neighbor backbone carbons (the carbon to which the ring is attached, and the carbon to which the next ring is attached).

**Figure 5:**
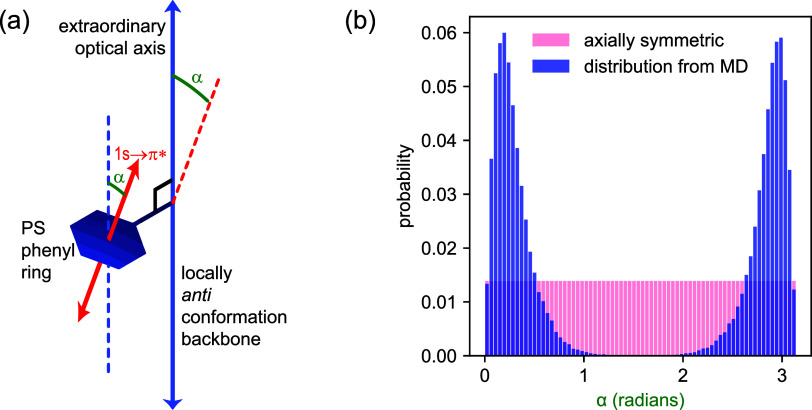
Distribution of angle between PS aromatic ring normal and polymer backbone obtained from atomistic calculations, described by (a) a schematic of a single phenyl ring attached to a locally *anti* PS backbone defining the angle α between phenyl ring normal and the backbone vector, and (b) a histogram of the calculated distribution in terms of α. We assume this distribution of orientation angles relative to the backbone is the same throughout our system.

In the initial configuration, all these angles were zero; after 50 ns of equilibration as described above, the distribution is nearly symmetric (see Figure 5), indicating that all rings have had time to “flip over”, i.e., to rotate by 180°. Figure 5 displays the resulting probability distribution, which is strongly peaked at angles near 0 and 180°, consistent with the expectation that for steric reasons, the rings tend to be oriented with their normals parallel (or antiparallel) to the backbone. The bimodal distribution observed in Figure 5 is in excellent agreement with deuterium NMR studies of deuterated polystyrene. Such studies have shown that the phenyl rings do not sample all orientations uniformly but instead undergo a bimodal equilibrium libration with occasional π-flips.^63^ The principal consequence of this intramolecular conformation preference specific to PS is that the 1s → π* resonance direction is a much stronger reporter of backbone orientation than we appreciated in our earlier axially symmetric assumption. The more strongly dichroic/birefringent uniaxial index of refraction would be expected to require a smaller orientation extent to produce the same amount of P-RSoXS pattern anisotropy. The uniaxial refractive index tensor with new ordinary and extraordinary components reflecting the distribution in Figure 5 is shown in Figure S2. The extraordinary and ordinary directions relative to the molecule have not changed from our earlier description, but the difference between the extraordinary and ordinary refraction indices is now larger. We expect this index of refraction to correctly describe PS soft X-ray optical properties for any PS-containing system in which no extreme stretching of the polymer backbone is observed or expected.

**Figure 6:**
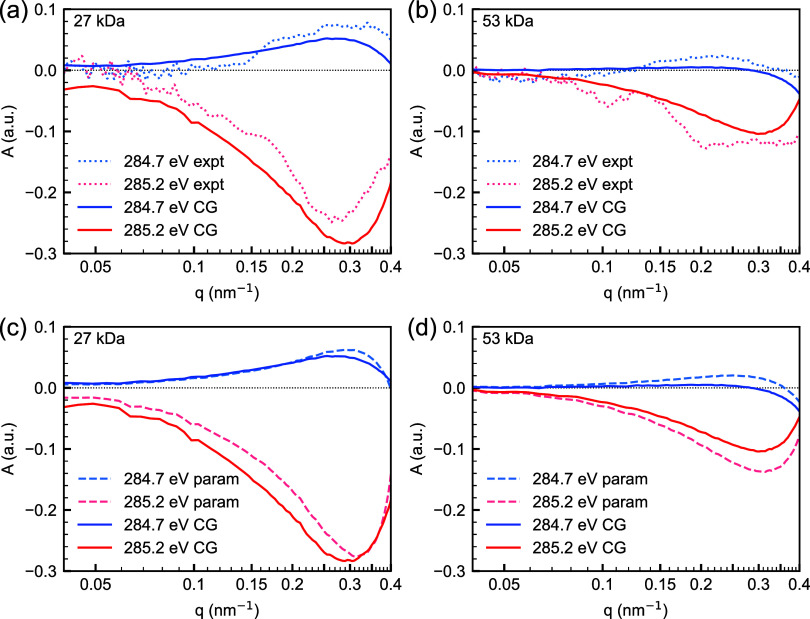
Experimental (expt) (dotted line) vs simulated (CG) (solid line) *q*-dependence of anisotropy parameter *A*(*q*) at 284.7 and 285.2 eV for the (a) AuPS27, and (b) AuPS53 system assuming radial chains. The uniaxial soft X-ray index of refraction used for the simulation was derived from the PS aromatic ring conformation distribution in the corona shown in Figure 5. Comparison of simulation results from coarse-grained morphology (CG, solid lines) and parametric model fits^19^ (param, dashed line) for (c) AuPS27, and (d) AuPS53 systems. For definition of *A*(*q*), see the Methods section.

We then resimulate the P-RSoXS from the same CG-based model described previously, but using this detailed description of the anisotropic refractive index of polystyrene from atomistic calculation, and we now achieve excellent agreement between the simulated and experimental *A*(*q*), as shown in Figure 6. The sum of squared errors (SSE) was calculated to determine the quality of fit between the experiment and the simulations, with details given in Table 1. Overall, the CG results have only slightly higher SSE compared to our earlier three-variable parametric fit; the major contribution to the SSE comes from the 285.2 eV data, which is also the energy where |*A*(*q*)| is maximum. The SSE of the high graft density sample (AuPS27) is considerably higher than the low graft density sample (AuPS53), likely due to noise from the low level of anisotropy.

**Table 1:** Sum of Squared Error (SSE) between Experiment and Simulations (CG and Parametric Models) for the AuPS27 and AuPS53 Samples, Computed at the Two Energies −284.7 and 285.2 eV^a^

	SSE	
sample	CG	parametric
AuPS27	2.94 (2.49)	1.66 (1.28)
AuPS53	1.05 (0.89)	0.79 (0.63)

a The numbers in parentheses are SSE values calculated at 285.2 eV, where |*A*(*q*)| is maximum.

Considering that there are no fitting parameters in the CG-based model, the quality of fit is indeed remarkable. Only a narrow window of real-space orientation characteristics can come close to fitting the magnitude and shape of the energy-dependent *A*(*q*) because it is extremely sensitive to the details of the spatial distribution of PS orientation around the nanoparticle. As illustrated in Figures S3–S5, subnanometer differences in the dimensions of the oriented corona profoundly affect agreement between simulation and experiment. And yet, the peak position and rates of rise/fall of *A*(*q*) for the CG-based model are an even better match to the experiment than our previously considered parametric model for both samples. The latter consistently overestimated the location of the peak *A*(*q*) as well as the decay of *A*(*q*). Our parametric model was constrained by a continuum mathematical description of orientation and its decay, whereas the CG model has no such constraints. The CG simulations capture well the thickness of the oriented region and the decay of that orientation of the CPB region of the brush for both cases. The major discrepancy observed between the experiment and simulations is in the magnitude of *A*(*q*), and it is stronger for the higher graft density sample. It should be noted here that the CG model does not include any free chains; (i.e., all the polymer chains in the CG model are grafted chains), whereas in the experimental sample, a nontrivial fraction (≈10% by volume or more) of the polymer exists as free (nongrafted, less oriented) chains. Therefore, the CG model overestimates the volume fraction of the oriented corona in the sample. Specifically, the thiol-based chemistry used to prepare the particles results in a significant fraction of demixed PS chains^64^ in the high graft density system. There is a remaining assumption in the conversion of CG results to the P-RSoXS simulation model that the chains within each voxel are best described by a radial director (described by radial Euler angles) with a degree of common bond orientation (S). More information could be conveyed from the CG model into the NRSS simulation with a voxel-specific director that is not strictly radial, but determining the Euler angles that preserve the greatest information requires numerical solution approaches within each voxel. A more faithful conversion of the CG results could also be achieved with a biaxial description or a full nine-element tensor index of refraction. Finally, a smaller voxel size could be combined with the above approaches to convey yet more detail from the model. These approaches to more information-rich optical descriptions would be significantly more computationally intensive to prepare and simulate, but might be approached in future efforts. A combination of the above factors is the most probable cause of the higher SSE for the AuPS27 sample.

**Figure 7:**
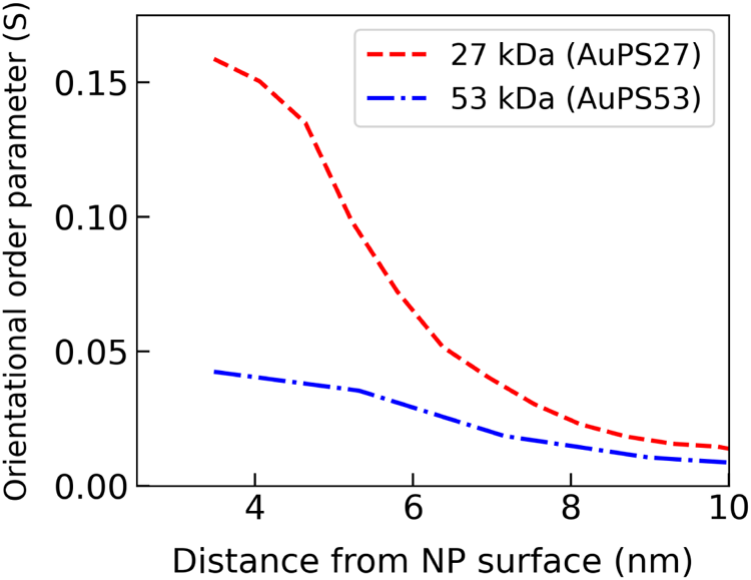
Radial distribution of order parameter as function of distance from particle surface obtained from radially averaged profiles of 3D AuPS27 and AuPS53 particle stamps.

The models for the two systems (AuPS27 and AuPS53) are summarized in Figure 7. Compared to AuPS27, the AuPS53 order parameter is lower and the oriented region thickness is slightly larger. The rates of decay of S are also different for the two samples, the decay in AuPS27 being ≈4× faster compared to that in AuPS53. The above results are broadly similar to what we have observed using a parametric model.^19^ The lower S in AuPS53 indicates a substantially reduced amount of overall chain orientation, which is consistent with its graft density being roughly half of that of AuPS27.

The reconciliation of chain orientation in polymer-grafted nanoparticles between coarse-grained models and P-RSoXS measurements further validates some of the deepest assumptions underlying CG modeling, particularly that an atomistic polymer backbone that is highly constrained by its sp^3^ bond rotations can be coarsened, with few free parameters and with flexible bonds, to beads at about the Kuhn length scale, while retaining chain conformational properties above that scale. Our P-RSoXS experiment interrogates the underlying, constrained bond system, whereas the P-RSoXS model that accurately represents them is derived from the springs of a CG nearly freely jointed chain (with a slight stiffness added to reproduce entanglement density relative to Kuhn length). The agreement we see here between experiment and model reveals that the (second) moments of those orientation distributions, and the spatial heterogeneity of those moments at the 2.5 nm nanometer scale, are nearly identical. The freely jointed chain CG model thus accurately describes chain stretching in this PGN system.

## Conclusions

We find excellent agreement between coarse-grained modeling and P-RSoXS measurements of spatially heterogeneous chain stretching in polystyrene-grafted gold nanoparticles. Agreement was found using data fusion approaches to build real-space P-RSoXS simulations that incorporated CG descriptions, yielding virtual instrument results nearly identical to experimentally collected P-RSoXS patterns. Application of the approach was demonstrated across two systems with different graft densities and chain lengths. Reconciliation of CG models and experimental results required challenging our previous assumption about polystryene phenyl ring conformation relative to the PS chain backbone. A new conformation based on atomistic modeling reveals a significant orientation preference to the polystryene phenyl ring that is similar to polystyrene conformation in isotactic and syndiotactic polystyrene crystals. The excellent agreement between coarse-grained modeling results and P-RSoXS measurements validates some of the deepest assumptions behind CG modeling and provides assurance that the method accurately describes chain stretching in confined systems. The unique bond orientation sensitivity of the P-RSoXS method and its intrinsically nanoscale resolution make it a powerful experimental companion to soft material simulation efforts. Simulation of P-RSoXS via virtual instrument from a high-resolution real-space description provides a flexible and accessible platform that faithfully preserves and incorporates the rich detail available from simulations in reconciliations with experiment. We expect paradigms that combine P-RSoXS with soft material simulation campaigns of many types to proliferate in the future as the technique expands to include broader classes of materials and frontiers in nanoscale chain stretching phenomena.

## Methods

### Molecular Dynamics Simulations

As in previous work by Ethier et al.^65,66^ we create polymer-grafted nanoparticles using spherical nanoparticles with a Kremer-Grest type of bead–spring model^18^ for polymers grafted to their surface at randomly selected points. Specifically, the linear graft chains are composed of monomers with Lennard-Jones (LJ) diameter σ and mass m, with the first monomer (termed the graft bead) rigidly attached to the nanoparticle surface with diameter 30σ.^65,66^ Very similar coarse-grained models of PGNs have been used successfully by multiple other groups, including to study bulk PGNs neat^39−42,67^ and in matrix polymer.^40,43,44^ These models have proven to be practical tools to look at assemblies of PGNs in the melt-like^66,67^ and glassy^45,68^ state, and across a variety of mechanical loading^40,42,45,68^ conditions.

We build monolayers by placing two nanoparticles in a hexagonal unit cell above a smooth wall, whose potential form is derived considering it is composed of a continuum of 12–6 LJ monomers of size σ. To represent a monolayer, which extends macroscopically in the *x* and *y* (in-plane) dimensions, but is only one nanoparticle layer thick, the simulation box is periodic in the transverse directions to the wall (*x* and *y*), but not *z*. The +*z* boundary is ‘shrink wrapped’, and adjusts to the size of the system (but does not actually confine the chains), whereas the −*z* boundary (representing a substrate) is fixed in place, and includes a wall potential. Particles and monomers are not allowed inside the wall, but are able to go any distance above it. Considering periodic images, a two-dimensional hexagonal lattice of polymer-grafted nanoparticles is formed. In contrast, using three-dimensional periodic boundaries would represent an element of a bulk system, which is not the system of interest.

We set the chain stiffness via an angle potential described below, and select its strength and the mapping to real length so that the volume occupied by an entanglement strand in a bulk homopolymer of our model matches that of polystyrene (meaning the entanglement densities will match). Specifically, we compared literature data (entanglement molecular weight and density) for polystyrene from Fetters^69^ with simulation data available for reference homopolymers of length *N* = 500 from prior unpublished work of Ethier. These simulations are used to get density and entanglement length via the modified S-coil estimator of Hoy.^70^ Further details describing these bulk homopolymer simulations and the selection of angle stiffness can be found in the Supporting Information, where the final length mapping of 0.67 nm/σ is explained in detail. This mapping implies that each coarse-grained monomer bead corresponds to roughly 2 polystyrene monomers.

Using this mapping, a nanoparticle size of 30σ is selected to roughly match the mean size of particles in the experimental samples (about 20 nm). There are two conditions of interest: (1) 27 kDa grafts at a graft density of 1.2 chains/nm^2^, and (2) 53 kDa grafts at a density of 0.6 chains/nm^2^; these conditions are chosen such that (1) and (2) contain the same amount of polymer. In this model these conditions are approximated by (1) 135 bead length grafts with graft density 0.27 chains/σ^2^, and (2) 270 bead length grafts with graft density 0.54 chains/σ^2^. The model particles and graft lengths are perfectly monodisperse for each system.

All other model details follow prior work and are briefly reviewed here. Beads are bonded along the chain using finitely extensible nonlinear elastic (FENE) bond potentials of the form
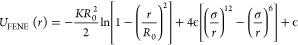
1with standard parameters *K* = 30ϵ/σ^2^, and *R*_0_ = 1.5σ. To better map to the polystyrene chains in the experimental samples as noted above, the chains were made relatively stiffer using an angle bending potential of the form
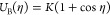
2where η is the angle between any three consecutive beads along the backbone; this common form was used in some of Ethier’s prior work,^65^ and by Kumar and co-workers in similar PGN simulations.^43^ The prefactor is chosen to be *K* = 0.5*k*_B_*T* as described further in the Supporting Information.

All other pairwise interactions are of standard 12–6 LJ form, cut and shifted to 0 at a distance of 2.5σ, and all monomer–monomer interaction strengths are equivalent (ϵ = 1*k*_B_*T*). However, the LJ potential used for monomer-nanoparticle interactions is shifted radially from the center of the nanoparticle (by 14.5σ), such that beyond the hard core, the nanoparticle-monomer interaction has the same strength and range as a monomer–monomer interaction.

Interactions with the wall are handled via a 9–3 LJ potential, cut and shifted to 0 at a distance of 5.5σ, which approximates the interaction of a single monomer with a perfectly smooth wall composed of a continuum of 12–6 LJ monomers of size σ. The wall-monomer and wall-nanoparticle interaction strengths are set to 3.5 *k*_B_*T*, which was found to produce stable monolayers in prior work.^66^

To initialize the simulations, graft beads are first placed randomly on the surface of the nanoparticle, and the chain is then created as a random walk (except disallowing crossing into the nanoparticle or wall). The initial size of the box is chosen to accommodate the excluded volume of the two nanoparticles plus the volume required for all monomers at a meltlike density of 0.89 beads/σ^3^. Relative side lengths are chosen to be appropriate for hexagonal packing. After the chains are created as random walks, monomer overlaps are reconciled using a brief simulation period with soft repulsive potentials and a limited maximum per step bead displacement.

After a brief further simulation with the full potentials at constant volume and energy with small timesteps and limited maximum per step displacement, each system is simulated with a Nóse-Hoover thermostat and barostat at a constant pressure of 0 and temperature of 1 in reduced units, with damping parameters 100 and 1.0 τ, respectively, and a time step of 0.01 τ where τ is the standard LJ unit of time, as in ref (66). The barostat is applied in a coupled fashion in the *x* and *y* directions (keeping the fixed side length ratio consistent with hexagonal packing) and not in the *z* direction (where particles are not constrained in the positive *z* direction, implying a vacuum above the film). Ten million steps of these dynamics are run (10^5^ τ), with 100 equally spaced frames from the last million steps used to produce orientation profiles. Each nanoparticle and its graft beads (the first bead of each grafted chain) is treated as a rigid body. Simulations are performed using the open source molecular dynamics (MD) software Large-scale Atomic/Molecular Massively Parallel Simulator (LAMMPS).^71^

### Coarse-Grained Chain Orientation Profiles

The NRSS framework^50^ simulates P-RSoXS patterns based on real-space voxel-style models. NRSS models are typically micron-scale representations with voxel dimensions on the order of single nanometers; our voxel size for this PGN study is (2.5 nm)^3^, somewhat larger than the diffraction limit across the carbon K-edge of (1.5 to 2) nm. To translate our preliminary 3D coarse-grained orientation profiles for the grafted chains (used to generate particle “stamps” in the final model) into NRSS model voxels, we first reduce the N grafted monomer or bead coordinates in each frame to a series of N-1 bond vectors. These bond vectors are then assigned to one of M 3D voxels of size (2.5 nm)^3^. Unit length radial directors from the centers of the nearest nanoparticle to the center of the voxel are calculated. As a preliminary estimate of chain orientation, the second Legendre polynomial
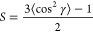
3describes a scalar measure of orientation extent in the voxel where *S* is an orientational order parameter and γ is the angle between the CG bond orientation and the radial director; *S* is averaged over all bond vectors within the voxel. We note that this process assumes that the second moment of the orientation distribution is defined by a radial director. This method does discard information about the orientation distribution of bonds on the subvoxel level, but it is a simplification necessary to represent the voxel with a single orientation of uniaxial indices of refraction having magnitude *S*. This process produces a 3D field estimating the ordering of bead–spring chains relative to the nanoparticle’s surface normal. Note that the CG models used here do not have explicit repeat unit chemistries or phenyl moieties. The field calculated here describes the orientation distribution of the backbone of the chain of CG beads, a larger length scale abstraction of the underlying molecular subunits. The results are averaged over the 100 frames over the last 10^4^ τ of the simulation run to generate preliminary orientation profiles and particle stamps.

### Mean Square Internal Distances

In addition to orientation profiles, chain conformations are characterized by finding mean squared internal distances as a function of chemical distance, *n* = *j* – *i*, where *j* and *i* are beads on a grafted chain. For each frame, we find chain-averaged pairwise distances separating the first bead (*i* = 1) on the chain from bead *j* = *n* + 1 according to
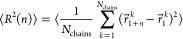
4where the brackets denote averaging over 100 frames in time, covering the last million steps (10^4^ τ). There are 1526 grafted chains for AuPS27 (the low graft density system), and 3052 chains for AuPS53 (the high graft density system). The maximum chemical distance is *n* = *N* – 1, where *N* = 270 beads for AuPS27, and *N* = 135 beads for AuPS53. All squared distances are converted to units of squared nanometers using the 0.67 nm/σ mapping, and then normalized by dividing eq 4 by chemical distance, *n*.

### Persistence Length

The decorrelation of bond vectors (i.e., vectors connecting adjacent beads on the grafted chains) separated by *n* bonds is used as a secondary measure of grafted chain orientation. We start by reducing each grafted chain to a series of unit bond vectors (i.e., each vector is scaled by the bond length). The correlation function, ⟨*b*_1_ · *b*_*n*_⟩, is found by dotting the unit director connecting the first bead on the chain with a bond vector *n* bonds farther down the chain. This process is repeated starting from the opposite end (the free end) of the chain, ⟨*b*_*N*_ · *b*_*N*–*n*_⟩. The averaging applied is identical to that used when calculating mean square internal distances. This correlation function is the standard for calculating persistence length, and is typically found by fitting to a simple exponential decay.

### Scattering Simulations

P-RSoXS simulations were performed using the NRSS, a recently developed virtual instrument platform.^50,72^ The overall morphology contains 32 × 512 × 512 voxels (as *Z* × *Y* × *X* dimensions) with each voxel cube having 2.5 nm sides and three component materials. The energy range used was 280 to 290 eV in steps on 0.1 (total 101 energies). Each voxel had 4 features^72^ associated with each component:

1. *v*_frac_: fraction of volume occupied by the component, and 0 ≤ *v*_frac_ ≤ 1. A morphology class method was used to check that the sum of all volume fractions of all the components equal 1.0.
2. *S*: orientational order parameter, which is a measure of the degree of alignment of the component in a voxel. Values of *S* fall in the range 0 ≤ *S* ≤ 1 where *S* = 0 indicates an isotropic condition and *S* = 1 indicates complete alignment with the radial director. The radial director is defined by the Euler angles θ and ϕ (defined below). The orientational order parameter is conceptually similar to the relative volume fraction (*s*) of the component that is perfectly oriented, and, when multiplied by *v*_frac_, yields the absolute volume fraction of perfectly oriented material in a voxel.
3. θ: Second Euler angle in the ZYZ Euler formalism; rotation of the extraordinary optical axis about the original *y* axis.
4. ϕ: Third Euler angle in the ZYZ Euler formalism; rotation of the extraordinary optical axis about the *z* axis.

NRSS simulations were performed in a Python environment on an NVIDIA Quadro A6000 GPU with 48 GB of GDDR6 global memory.

### Experimental Section

The sample preparation, characterization, and P-RSoXS experimental details are given in detail elsewhere^19,64^ and briefly outlined here.

#### Sample Preparation

The PGN dispersions were flow-coated on backside-patterned silicon/silicon nitride (56 nm) substrates. The blade height and angle were 250 μm and 5°, respectively. The substrate was placed on a translational stage that was moved at a constant speed of 10 mm/s, and the coated film was air-dried. In order to back-etch the silicon backing of the windows, the coated films were put in a custom polyether ether ketone (PEEK) wet etch holder and submerged in a KOH bath to expose the nitride windows.^73^ After the etch was complete, the wafers were taken out of the holder and air-dried.

#### Polarized Resonant Soft X-ray Scattering (P-RSoXS)

P-RSoXS experiments were done in transmission geometry at the Advanced Light Source (ALS) beamline BL 11.0.1.2.^74^ The 2-D scattering patterns were collected using an in-vacuum charge-coupled device (CCD) detector (PI-MTE, Princeton Instruments) having 2048 × 2048 pixels with a pixel size of 27 μm. Scattering data were collected at different energies across the C K-edge covering the range 270 to 290 eV with vertical and horizontal incident X-ray polarizations (relative to laboratory frame). The scattering data were analyzed using a recently developed python-based analysis package.^75^

## Supporting Information Available

The SI contains the optical constants used in P-RSoXS simulations for both axially symmetric and atomistically derived phenyl ring conformations. It presents detailed parameter sweeps demonstrating the sensitivity of anisotropy (A) to variations in canopy thickness, near-surface orientation strength, and decay order of the orientational order parameter. The document also provides the methodology and justification for the selection of chain stiffness in coarse-grained models, including mapping to real units based on entanglement characteristics of polystyrene. Finally, it describes the boundary conditions and simulation cell dimensions used in modeling PGN monolayers (PDF)

## References

[ref1] Tobolsky A.; Eyring H. Mechanical Properties of Polymeric Materials. J. Chem. Phys. 1943, 11, 125–134. 10.1063/1.1723812.

[ref2] Rouse P. E. A Theory of the Linear Viscoelastic Properties of Dilute Solutions of Coiling Polymers. J. Chem. Phys. 1953, 21, 1272–1280. 10.1063/1.1699180.

[ref3] Doi M.; Edwards S. The Theory of Polymer Dynamics. In International Series of Monographs on Physics; Clarendon Press, 1988.

[ref4] De Gennes P. G. Dynamics of Entangled Polymer Solutions. I. The Rouse Model. Macromolecules 1976, 9, 587–593. 10.1021/ma60052a011.

[ref5] De Gennes P. G. Dynamics of Entangled Polymer Solutions. II. Inclusion of Hydrodynamic Interactions. Macromolecules 1976, 9, 594–598. 10.1021/ma60052a012.

[ref6] Pincus P. Excluded Volume Effects and Stretched Polymer Chains. Macromolecules 1976, 9, 386–388. 10.1021/ma60051a002.

[ref7] Bonart R. Thermoplastic elastomers. Polymer 1979, 20, 1389–1403. 10.1016/0032-3861(79)90280-5.

[ref8] Hiss R.; Hobeika S.; Lynn C.; Strobl G. Network Stretching, Slip Processes, and Fragmentation of Crystallites during Uniaxial Drawing of Polyethylene and Related Copolymers. A Comparative Study. Macromolecules 1999, 32, 4390–4403. 10.1021/ma981776b.

[ref9] Hadziioannou G.; Patel S.; Granick S.; Tirrell M. Forces between surfaces of block copolymers adsorbed on mica. J. Am. Chem. Soc. 1986, 108, 2869–2876. 10.1021/ja00271a014.

[ref10] Milner S. T.; Witten T. A.; Cates M. E. Theory of the grafted polymer brush. Macromolecules 1988, 21, 2610–2619. 10.1021/ma00186a051.

[ref11] Kraus J.; Müller-Buschbaum P.; Kuhlmann T.; Schubert D. W.; Stamm M. Confinement effects on the chain conformation in thin polymer films. Europhys. Lett. 2000, 49, 210 10.1209/epl/i2000-00135-4.

[ref12] Daoud M.; Cotton J. P. Star Shaped Polymers - a Model for the Conformation and Its Concentration-Dependence. J. Phys. 1982, 43, 531–538. 10.1051/jphys:01982004303053100.

[ref13] Zhang R.; Lee B.; Stafford C. M.; Douglas J. F.; Dobrynin A. V.; Bockstaller M. R.; Karim A. Entropy-driven segregation of polymer-grafted nanoparticles under confinement. Proc. Natl. Acad. Sci. U.S.A. 2017, 114, 2462–2467. 10.1073/pnas.1613828114. 28228522 PMC5347555

[ref14] Wei Y.; Chen Q.; Zhao H.; Duan P.; Zhang L.; Liu J. Conformation and Dynamics along the Chain Contours of Polymer-Grafted Nanoparticles. Langmuir 2023, 39, 11003–11015. 10.1021/acs.langmuir.3c01238. 37493597

[ref15] Sunday D. F.; Dolejsi M.; Chang A. B.; Richter L. J.; Li R.; Kline R. J.; Nealey P. F.; Grubbs R. H. Confinement and Processing Can Alter the Morphology and Periodicity of Bottlebrush Block Copolymers in Thin Films. ACS Nano 2020, 14, 17476–17486. 10.1021/acsnano.0c07777. 33225683

[ref16] Li L.; Qiang Z.; Chen X.; Jin K.; Wang M.; Torkelson J. M. Impact of bottlebrush chain architecture on Tg-confinement and fragility-confinement effects enabled by thermo-cleavable bottlebrush polymers synthesized by radical coupling and atom transfer radical polymerization. J. Polym. Sci. 2020, 58, 2887–2905. 10.1002/pol.20200537.

[ref17] Chan J. M.; Kordon A. C.; Zhang R.; Wang M. Direct visualization of bottlebrush polymer conformations in the solid state. Proc. Natl. Acad. Sci. U.S.A. 2021, 118, e2109534118 10.1073/pnas.2109534118. 34599105 PMC8501853

[ref18] Kremer K.; Grest G. S. Dynamics of entangled linear polymer melts: A molecular-dynamics simulation. J. Chem. Phys. 1990, 92, 5057–5086. 10.1063/1.458541.

[ref19] Mukherjee S.; Streit J. K.; Gann E.; Saurabh K.; Sunday D. F.; Krishnamurthy A.; Ganapathysubramanian B.; Richter L. J.; Vaia R. A.; DeLongchamp D. M. Polarized X-ray scattering measures molecular orientation in polymer-grafted nanoparticles. Nat. Commun. 2021, 12, 4896 10.1038/s41467-021-25176-4. 34385430 PMC8361200

[ref20] Kumar S. K.; Benicewicz B. C.; Vaia R. A.; Winey K. I. 50th Anniversary Perspective: Are Polymer Nanocomposites Practical for Applications?. Macromolecules 2017, 50, 714–731. 10.1021/acs.macromol.6b02330.

[ref21] Sunday D.; Curras-Medina S.; Green D. L. Impact of Initiator Spacer Length on Grafting Polystyrene from Silica Nanoparticles. Macromolecules 2010, 43, 4871–4878. 10.1021/ma1004259.

[ref22] Tsukagoshi T.; Kondo Y.; Yoshino N. Protein adsorption on polymer-modified silica particle surface. Colloids Surf., B 2007, 54, 101–107. 10.1016/j.colsurfb.2006.10.004. 17118630

[ref23] García A. J. Polymers for Regenerative Medicine; Springer Berlin Heidelberg: Berlin, Heidelberg, 2006; pp 171–190.

[ref24] Napier M. E.; DeSimone J. M. Nanoparticle Drug Delivery Platform. Polym. Rev. 2007, 47, 321–327. 10.1080/15583720701454999.

[ref25] Delcamp J. H.; Martin K. L.; Posey N. D.; Acord K. A.; Thompson C. M.; Dickerson M. B. Preceramic Polymers Grafted to SiO2 Nanoparticles via Metal Coordination Pyrolyzing with High Ceramic Yields: Implications for Aerospace Propulsion and Biomedical Coatings. ACS Appl. Nano Mater. 2023, 6, 3661–3674. 10.1021/acsanm.2c05394.

[ref26] Chancellor A. J.; Seymour B. T.; Zhao B. Characterizing polymer-grafted nanoparticles: From basic defining parameters to behavior in solvents and self-assembled structures. Anal. Chem. 2019, 91, 6391–6402. 10.1021/acs.analchem.9b00707. 31013073

[ref27] Kumar S. K.; Jouault N.; Benicewicz B.; Neely T. Nanocomposites with Polymer Grafted Nanoparticles. Macromolecules 2013, 46, 3199–3214. 10.1021/ma4001385.

[ref28] Pyun J.; Matyjaszewski K. Synthesis of Nanocomposite Organic/Inorganic Hybrid Materials Using Controlled/“Living” Radical Polymerization. Chem. Mater. 2001, 13, 3436–3448. 10.1021/cm011065j.

[ref29] Khani M. M.; Abbas Z. M.; Benicewicz B. C. Well-defined polyisoprene-grafted silica nanoparticles via the RAFT process. J. Polym. Sci., Part A: Polym. Chem. 2017, 55, 1493–1501. 10.1002/pola.28514.

[ref30] Naskar A. K.; Keum J. K.; Boeman R. G. Polymer matrix nanocomposites for automotive structural components. Nat. Nanotechnol. 2016, 11, 1026–1030. 10.1038/nnano.2016.262. 27920443

[ref31] Ohno K.; Morinaga T.; Takeno S.; Tsujii Y.; Fukuda T. Suspensions of silica particles grafted with concentrated polymer brush: A new family of colloidal crystals. Macromolecules 2006, 39, 1245–1249. 10.1021/ma0521708.

[ref32] Ohno K.; Morinaga T.; Takeno S.; Tsujii Y.; Fukuda T. Suspensions of silica particles grafted with concentrated polymer brush: Effects of graft chain length on brush layer thickness and colloidal crystallization. Macromolecules 2007, 40, 9143–9150. 10.1021/ma071770z.

[ref33] Dahal U.; Dormidontova E. E. Chain conformation and hydration of polyethylene oxide grafted to gold nanoparticles: curvature and chain length effect. Macromolecules 2020, 53, 8160–8170. 10.1021/acs.macromol.0c01499.

[ref34] Vogiatzis G. G.; Theodorou D. N. Structure of polymer layers grafted to nanoparticles in silica-polystyrene nanocomposites. Macromolecules 2013, 46, 4670–4683. 10.1021/ma400107q.

[ref35] Midya J.; Rubinstein M.; Kumar S. K.; Nikoubashman A. Structure of Polymer-Grafted Nanoparticle Melts. ACS Nano 2020, 14, 15505–15516. 10.1021/acsnano.0c06134. 33084300 PMC8056455

[ref36] Miller C. A.; Hore M. J. A. Simulation of the Coronal Dynamics of Polymer-Grafted Nanoparticles. ACS Polym. Au 2022, 2, 157–168. 10.1021/acspolymersau.1c00031. 36855522 PMC9954254

[ref37] Dodd P. M.; Jayaraman A. Monte carlo simulations of polydisperse polymers grafted on spherical surfaces. J. Polym. Sci., Part B: Polym. Phys. 2012, 50, 694–705. 10.1002/polb.23057.

[ref38] Ndoro T. V. M.; Voyiatzis E.; Ghanbari A.; Theodorou D. N.; Böhm M. C.; Müller-Plathe F. Interface of grafted and ungrafted silica nanoparticles with a polystyrene matrix: Atomistic molecular dynamics simulations. Macromolecules 2011, 44, 2316–2327. 10.1021/ma102833u.

[ref39] Midya J.; Cang Y.; Egorov S. A.; Matyjaszewski K.; Bockstaller M. R.; Nikoubashman A.; Fytas G. Disentangling the role of chain conformation on the mechanics of polymer tethered particle materials. Nano Lett. 2019, 19, 2715–2722. 10.1021/acs.nanolett.9b00817. 30913883 PMC6463242

[ref40] Goyal S.; Escobedo F. A. Structure and transport properties of polymer grafted nanoparticles. J. Chem. Phys. 2011, 135, 184902 10.1063/1.3657831. 22088076

[ref41] Yu J. W.; Yun H.; Lee W. B.; Kim Y. Two-Regime Conformation of Grafted Polymer on Nanoparticle Determines Symmetry of Nanoparticle Self-Assembly. Adv. Sci. 2024, 11, 2406720 10.1002/advs.202406720. PMC11422811 39073253

[ref42] Pal S.; Keten S. Micro-ballistic response of thin film polymer grafted nanoparticle monolayers. Soft Matter 2024, 20, 7926–7935. 10.1039/D4SM00718B. 39331362

[ref43] Koh C.; Grest G. S.; Kumar S. K. Assembly of polymer-grafted nanoparticles in polymer matrices. ACS Nano 2020, 14, 13491–13499. 10.1021/acsnano.0c05495. 33030334

[ref44] Meng D.; Kumar S. K.; Lane J. M. D.; Grest G. S. Effective interactions between grafted nanoparticles in a polymer matrix. Soft Matter 2012, 8, 5002–5010. 10.1039/c2sm07395a.

[ref45] Moussavi A.; Pal S.; Wu Z.; Keten S. Characterizing the shear response of polymer-grafted nanoparticles. J. Chem. Phys. 2024, 160, 134903 10.1063/5.0188494. 38573850

[ref46] Chancellor A. J.; Seymour B. T.; Zhao B. Characterizing Polymer-Grafted Nanoparticles: From Basic Defining Parameters to Behavior in Solvents and Self-Assembled Structures. Anal. Chem. 2019, 91, 6391–6402. 10.1021/acs.analchem.9b00707. 31013073

[ref47] Hore M. J. A. Polymers on nanoparticles: structure & dynamics. Soft Matter 2019, 15, 1120–1134. 10.1039/C8SM02110D. 30657158

[ref48] Kumar S. K.; Jouault N.; Benicewicz B.; Neely T. Nanocomposites with polymer grafted nanoparticles. Macromolecules 2013, 46, 3199–3214. 10.1021/ma4001385.

[ref49] Fernandes N. J.; Koerner H.; Giannelis E. P.; Vaia R. A. Hairy nanoparticle assemblies as one-component functional polymer nanocomposites: opportunities and challenges. MRS Commun. 2013, 3, 13–29. 10.1557/mrc.2013.9.

[ref50] usnistgov NRSS. 2024 https://github.com/usnistgov/NRSS. (accessed May 28, 2024).

[ref51] Humphrey W.; Dalke A.; Schulten K. VMD: Visual molecular dynamics. J. Mol. Graphics 1996, 14, 33–38. 10.1016/0263-7855(96)00018-5. 8744570

[ref52] Wessels M. G.; Jayaraman A. Computational Reverse-Engineering Analysis of Scattering Experiments (CREASE) on Amphiphilic Block Polymer Solutions: Cylindrical and Fibrillar Assembly. Macromolecules 2021, 54, 783–796. 10.1021/acs.macromol.0c02265.

[ref53] Stöhr J. NEXAFS Spectroscopy; Springer-Verlag, 1992; Vol. 25.

[ref54] Attwood D. T. Soft X-rays and Extreme Ultraviolet Radiation: Principles and Applications; Cambridge University Press, 2000.

[ref55] Wallace W. E.; Fischer D. A.; Efimenko K.; Wu W. L.; Genzer J. Polymer chain relaxation: Surface outpaces bulk. Macromolecules 2001, 34, 5081–5082. 10.1021/ma002075t.

[ref56] Zeghal M.; Deloche B.; Albouy P.-A.; Auroy P. Chain-segment order and dynamics in a grafted polymer melt: A deuterium NMR study. Phys. Rev. E 1997, 56, 5603 10.1103/PhysRevE.56.5603.

[ref57] Natta G.; Corradini P.; Bassi I. Crystal structure of isotactic polystyrene. Nuovo Cimento 1960, 15, 68–82. 10.1007/BF02731861.

[ref58] De Rosa C.; Guerra G.; Petraccone V.; Corradini P. Crystal structure of the *α*-form of syndiotactic polystyrene. Polym. J. 1991, 23, 1435–1442. 10.1295/polymj.23.1435.

[ref59] Chatani Y.; Shimane Y.; Ijitsu T.; Yukinari T. Structural study on syndiotactic polystyrene: 3. Crystal structure of planar form I. Polymer 1993, 34, 1625–1629. 10.1016/0032-3861(93)90319-6.

[ref60] Clancy T. C.; Jang J. H.; Dhinojwala A.; Mattice W. L. Orientation of Phenyl Rings and Methylene Bisectors at the Free Surface of Atactic Polystyrene. J. Phys. Chem. B 2001, 105, 11493–11497. 10.1021/jp011588e.

[ref61] Briggman K. A.; Stephenson J. C.; Wallace W. E.; Richter L. J. Absolute Molecular Orientational Distribution of the Polystyrene Surface. J. Phys. Chem. B 2001, 105, 2785–2791. 10.1021/jp0037495.

[ref62] Jorgensen W. L.; Maxwell D. S.; Tirado-Rives J. Development and Testing of the OPLS All-Atom Force Field on Conformational Energetics and Properties of Organic Liquids. J. Am. Chem. Soc. 1996, 118, 11225–11236. 10.1021/ja9621760.

[ref63] Spiess H. W. Molecular dynamics of solid polymers as revealed by deuteron NMR. Colloid Polym. Sci. 1983, 261, 193–209. 10.1007/BF01469664.

[ref64] Che J.; Park K.; Grabowski C. A.; Jawaid A.; Kelley J.; Koerner H.; Vaia R. A. Preparation of Ordered Monolayers of Polymer Grafted Nanoparticles: Impact of Architecture, Concentration, and Substrate Surface Energy. Macromolecules 2016, 49, 1834–1847. 10.1021/acs.macromol.5b02722.

[ref65] Ethier J. G.; Hall L. M. Modeling individual and pairs of adsorbed polymer-grafted nanoparticles: structure and entanglements. Soft Matter 2018, 14, 643–652. 10.1039/C7SM02116J. 29271451

[ref66] Ethier J. G.; Hall L. M. Structure and entanglement network of model polymer-grafted nanoparticle monolayers. Macromolecules 2018, 51, 9878–9889. 10.1021/acs.macromol.8b01373.

[ref67] Midya J.; Rubinstein M.; Kumar S. K.; Nikoubashman A. Structure of polymer-grafted nanoparticle melts. ACS Nano 2020, 14, 15505–15516. 10.1021/acsnano.0c06134. 33084300 PMC8056455

[ref68] Ethier J. G.; Drummy L. F.; Vaia R. A.; Hall L. M. Uniaxial deformation and crazing in glassy polymer-grafted nanoparticle ultrathin films. ACS Nano 2019, 13, 12816–12829. 10.1021/acsnano.9b05001. 31609111

[ref69] Fetters L. J.; Lohse D.; Richter D.; Witten T.; Zirkel A. Connection between polymer molecular weight, density, chain dimensions, and melt viscoelastic properties. Macromolecules 1994, 27, 4639–4647. 10.1021/ma00095a001.

[ref70] Hoy R. S.; Foteinopoulou K.; Kröger M. Topological analysis of polymeric melts: Chain-length effects and fast-converging estimators for entanglement length. Phys. Rev. E 2009, 80, 031803 10.1103/PhysRevE.80.031803. 19905139

[ref71] Thompson A. P.; Aktulga H. M.; Berger R.; Bolintineanu D. S.; Brown W. M.; Crozier P. S.; in’t Veld P. J.; Kohlmeyer A.; Moore S. G.; Nguyen T. D.; et al. LAMMPS-a flexible simulation tool for particle-based materials modeling at the atomic, meso, and continuum scales. Comput. Phys. Commun. 2022, 271, 108171 10.1016/j.cpc.2021.108171.

[ref72] Saurabh K.; Dudenas P. J.; Gann E.; Reynolds V. G.; Mukherjee S.; Sunday D.; Martin T. B.; Beaucage P. A.; Chabinyc M. L.; DeLongchamp D. M.; Krishnamurthy A.; Ganapathysubramanian B. *CyRSoXS*: a GPU-accelerated virtual instrument for polarized resonant soft X-ray scattering. J. Appl. Crystallogr. 2023, 56, 868–883. 10.1107/S1600576723002790. 37284258 PMC10241048

[ref73] Ren J.; Ocola L. E.; Divan R.; Czaplewski D. A.; Segal-Peretz T.; Xiong S.; Kline R. J.; Arges C. G.; Nealey P. F. Post-directed-self-assembly membrane fabrication for in situ analysis of block copolymer structures. Nanotechnology 2016, 27, 435303 10.1088/0957-4484/27/43/435303. 27659775

[ref74] Gann E.; Young A. T.; Collins B. A.; Yan H.; Nasiatka J.; Padmore H. A.; Ade H.; Hexemer A.; Wang C. Soft x-ray scattering facility at the Advanced Light Source with real-time data processing and analysis. Rev. Sci. Instrum. 2012, 83, 045110 10.1063/1.3701831. 22559579

[ref75] USNISTGOV/Pyhyperscattering: Tools for hyperspectral X-ray and neutron scattering data loading, reduction, slicing, and visualization. https://github.com/usnistgov/PyHyperScattering.

