# Supplementary material for: Reconciling Chain Orientation in Polymer-Grafted Nanoparticles between Coarse-Grained Models and Resonant Soft X-ray Scattering
Source: ACS Nano. 2025 Apr 17;19(16):15638–50. doi: 10.1021/acsnano.4c18022 (PMC12044698; doi:10.1021/acsnano.4c18022)
Supplement: Supplementary file 1 — nn4c18022_si_001.pdf [file nn4c18022_si_001.pdf]

Supporting Information
Reconciling chain orientation in polymer-grafted nanoparticles between coarse-grained models and resonant soft X-ray scattering

Subhrangsu Mukherjee,[†] Nicholas T. Liesen,[‡] Scott T. Milner,[¶] Lisa M. Hall,[§] and Dean M. DeLongchamp[*,†]

†*Materials Science and Engineering Division, Materials Measurement Laboratory, National Institute of Standards and Technology, Gaithersburg, Maryland 20899, United States*

‡*Physical and Life Sciences Directorate, Lawrence Livermore National Laboratory, Livermore, California 94550, United States*

¶*Department of Chemical Engineering, The Pennsylvania State University, University Park, Pennsylvania 16802, United States*

§*Department of Chemical and Biomolecular Engineering, The Ohio State University, Columbus, Ohio 43221, United States*

E-mail: dean.delongchamp@nist.gov

Optical constants used for simulations

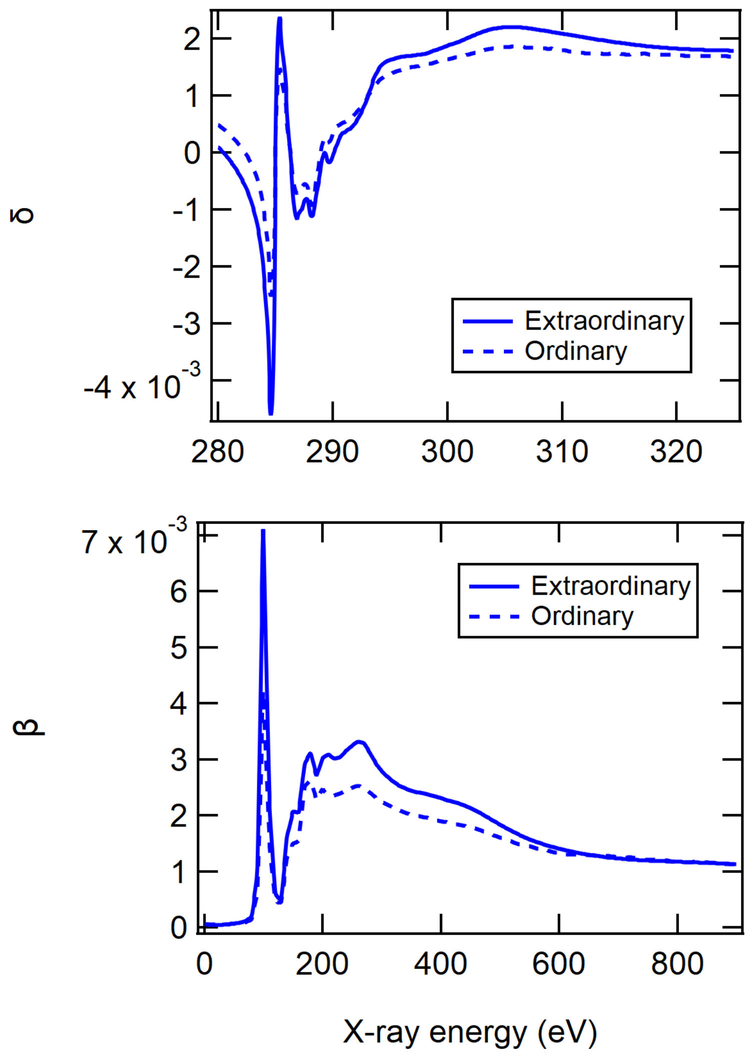

Figure S1: Real (δ) and imaginary (β) parts of the extraordinary and ordinary components of the complex dielectric tensor near the C K-edge for the "axially symmetric" molecular configuration.

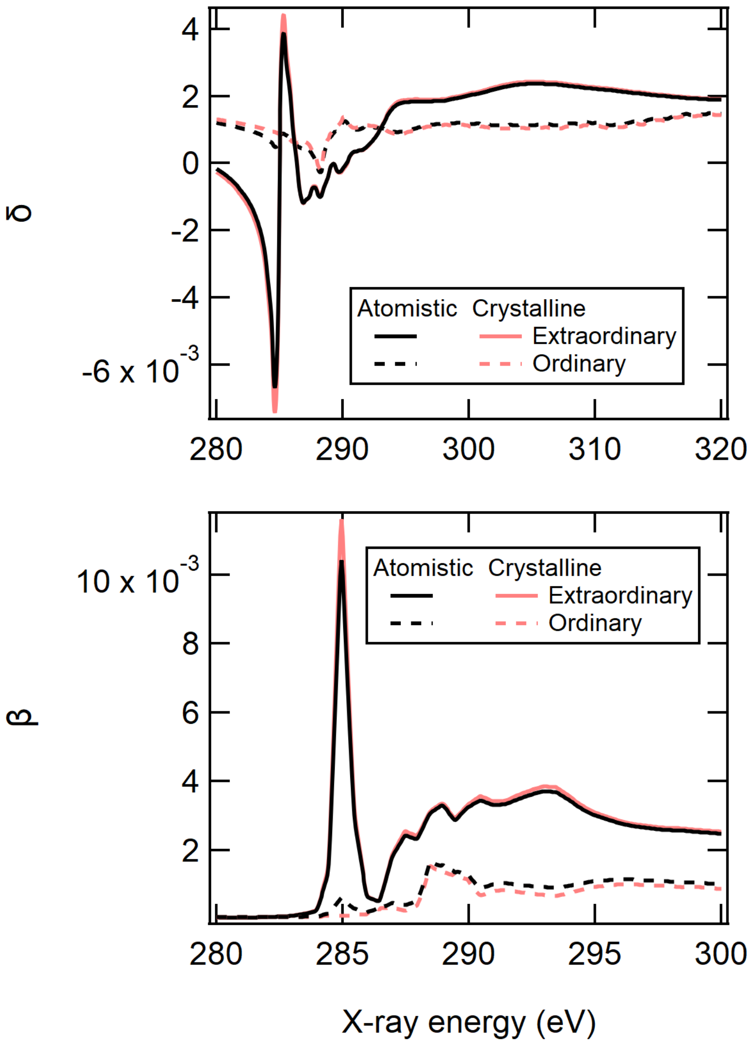

Figure S2: Real (δ) and imaginary (β) parts of the extraordinary and ordinary components of the complex dielectric tensor near the C K-edge for the molecular configuration obtained from atomistic calculations, and compared with the perfectly crystalline case.

Sensitivity of anisotropy to radial strength of molecular orientation

To demonstrate the significant sensitivity of the P-RSoXS measurement to the details of spatial heterogeneity of radial orientation in PGNs, we provide the following parameter sweeps of a parametric orientation distribution used in our earlier manuscript.[1] Even small variations in the parametric description of orientation decay in the oriented corona of the PGN exhibits significant effects in the shape of anisotropy (A) as a function of reciprocal space momentum transfer (q). These sweeps feature the PS index of refraction derived from the atomistic calculations presented in the body of the main manuscript, along with a re-tuned S_0 value; the best-fit value of the other parameters of our parametric description remain unchanged.

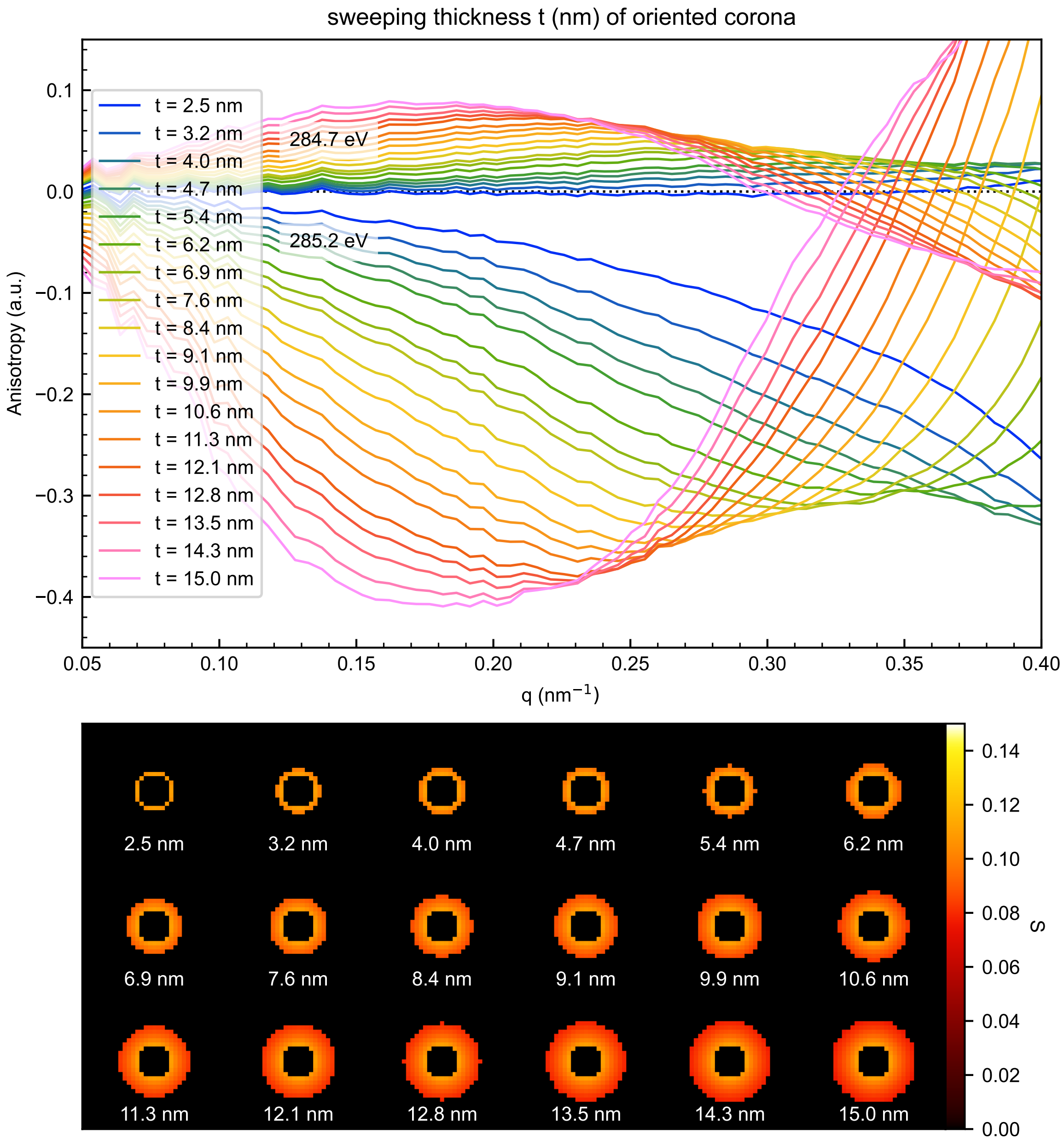

Figure S3: Variation in the anisotropy (A) vs. reciprocal space momentum transfer (q) as a function of the thickness of the anistropic canopy, at two key incident photon energies of 284.7 eV and 285.2 eV. The lower panel shows a cross-section example of the real-space orientation heterogeneity for a model single nanoparticle; simulations were for the full ensemble described by AFM data fusion.

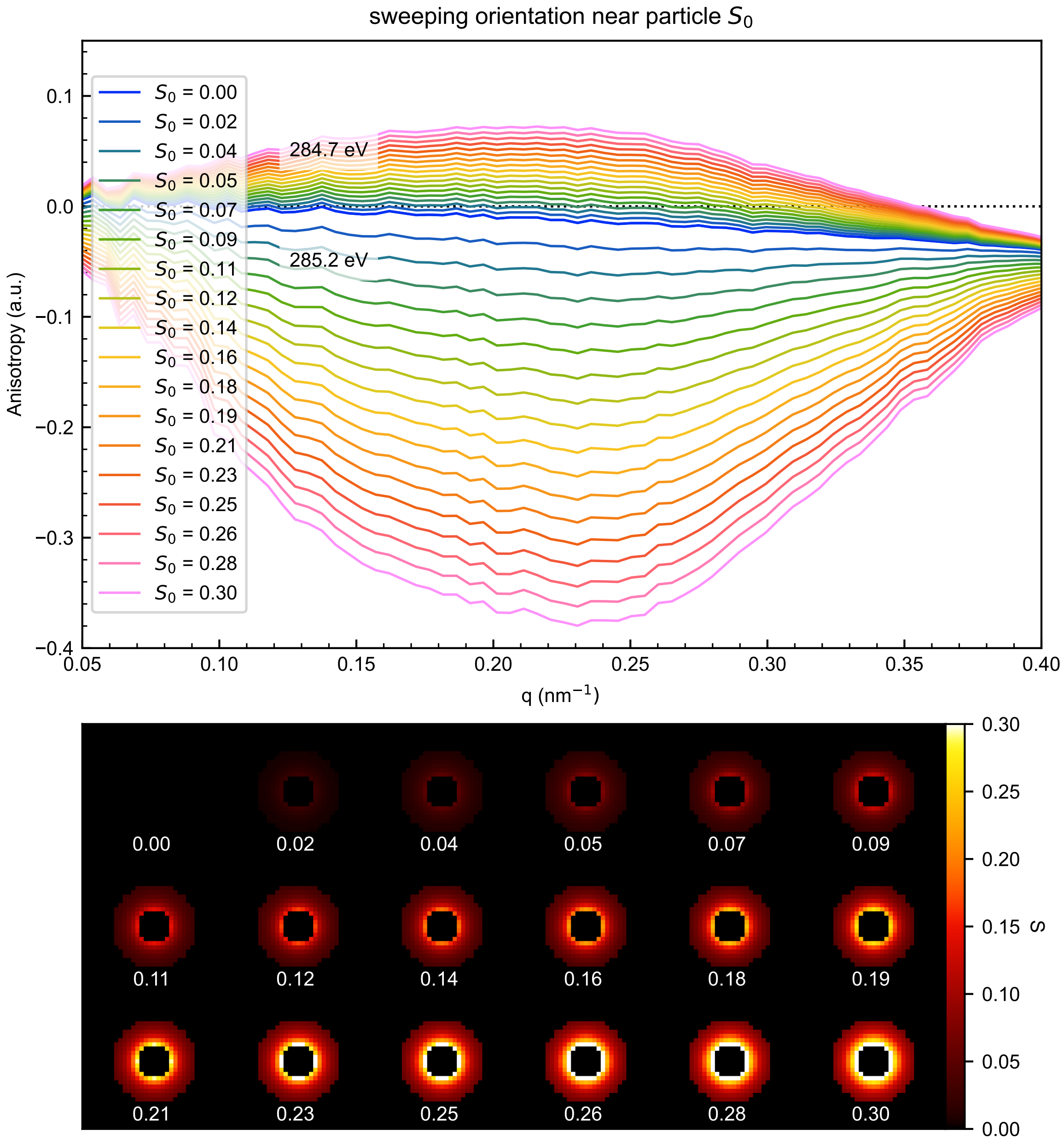

Figure S4: Variation in the anisotropy (A) vs. reciprocal space momentum transfer (q) as a function of the extent of orientation immediately next to the particle, at two key incident photon energies of 284.7 eV and 285.2 eV. The lower panel shows a cross-section example of the real-space orientation heterogeneity for a model single nanoparticle; simulations were for the full ensemble described by AFM data fusion.

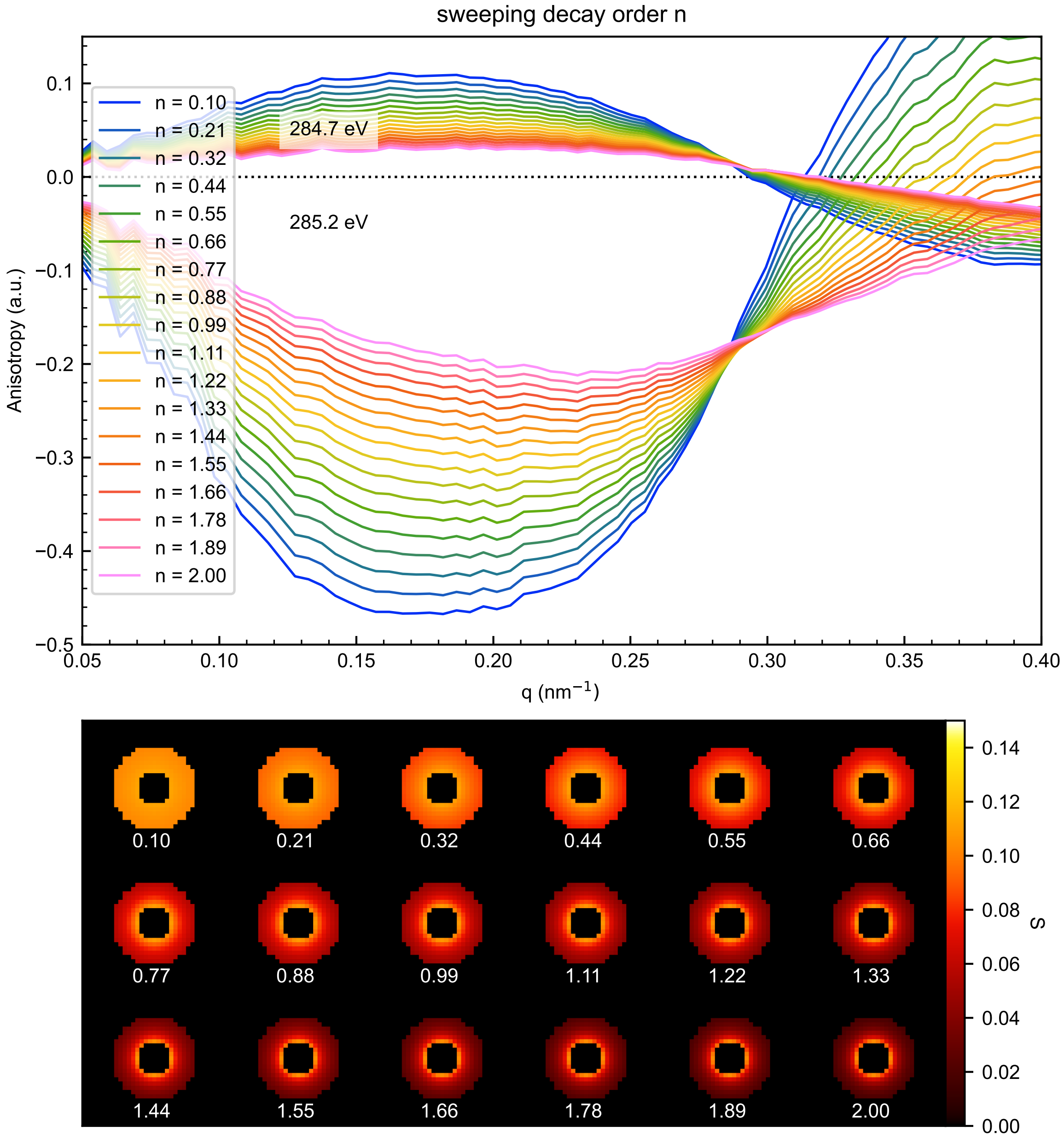

Figure S5: Variation in the anisotropy (A) vs. reciprocal space momentum transfer (q) as a function of order to which orientation decays as a function of distance, at two key incident photon energies of 284.7 eV and 285.2 eV. The lower panel shows a cross-section example of the real-space orientation heterogeneity for a model single nanoparticle; simulations were for the full ensemble described by AFM data fusion.

MD chain stiffness and mapping to real units

To better map to experimental samples with PS grafts, we added stiffness to our model chains by applying an angle potential of the form $K(1 + cos\theta)$ between every three consecutive beads along the backbone; this is a common form that was also used in some of some of Jeffrey Ethier's prior work.[2] The prefactor of the angle potential was chosen to be 0.5 kT as described below. To determine the appropriate stiffness, we used data from prior reference simulations of Jeffrey Ethier on bulk homopolymers that use the same model as graft chains (without nanoparticles or a surface). Specifically, data was available for chain length N=500 homopolymers for which 300 chains were initialized as random walks in a simulation box and were equilibrated with a Nosé-Hoover thermostat and barostat using similar conditions as in Ref. 3 but with double bridging Monte Carlo (MC) moves applied to allow chains to effectively cross and speed equilibration. These long chains were chosen to give an accurate value of the entanglement length. An additional simulation was then run without such moves, and the densities and primitive path data (using the Z1 algorithm)[4,5] were calculated from this simulation. For fully flexible chains, the initial equilibration time with MC moves was 350 million timesteps, and the simulation was run 20 million timesteps further before a data collection period of an additional 40 million timesteps. For accurate data at the chosen value of K=0.5, the simulation was run longer, specifically, 450 million timesteps for equilibration with MC moves, followed by 180 million timesteps of further equilibration, and data collection over 40 million timesteps. For a brief test at K=0.75 used for comparison, the system was equilibrated for 250 million timesteps with data collection for 10 million timesteps (due to the lower equilibration and data collection times, the entanglement value of this sample is known with less accuracy).

Using the average squared primitive path length $\langle L_{pp}^2 \rangle$ and end-to-end vector $\langle R_{ee}^2 \rangle$ length data from the above simulations, the entanglement length was calculated using the modified S-coil estimator of Hoy in Ref. 6. For the K=0, 0.5, and 0.75, the bead number densities ρ were 0.892, 0.890, and 0.889 σ^{-3}, $\langle R_{ee}^2 \rangle^{1/2}$ were 29.35, 31.02, and 32.19 σ, and the entangle-

ment lengths were 89, 68, and 58 beads, respectively. We used this data, along with the PS data of Fetters in Ref. 7 (density of 0.969 g/cm^3, entanglement molecular weight of 13,300 g/mol, and packing length of 0.395 nm) to map the model to real units. In the current PGN system, the volume taken up by the chains relative to the nanoparticle size is a key determinant of the interparticle spacing, and we also hope to capture the appropriate number of entanglements per volume. Other key features of chains are the extent to which the chains interpenetrate (related to their average R_{ee} relative to their length) and the packing length $p = \frac{N}{\rho\langle R_{ee}^2\rangle}$ (a measure of the effective thickness of chains related to entanglements). We therefore map to nm units by matching the volume taken up by one entanglement length of polymer (or entanglement density). Using the density and entanglement length values provided above, this yields a mapping of 0.61, 0.67, and 0.70 nm/σ with 1.4, 1.9, and 2.2 PS monomers represented by one bead, for K=0, 0.5, and 0.75, respectively. Using this mapping to nm along with the above densities and $\langle R_{ee}^2\rangle^{1/2}$ values yields a packing length of 0.398, 0.390, and 0.382 nm for K=0, 0.5, and 0.75, respectively. The experimental packing length of PS of 0.395 nm is between that calculated for K=0 and 0.5 samples using this method. Rather than choosing a more specific value of K to more exactly match packing length, we chose to apply the stiffness of K=0.5 for the current work. This value also has been used in some prior work and yields a favorable mapping of ∼2 PS monomers per bead, leading to less beads required in the simulation relative to a fully flexible model. Using the stiffness of K=0.5 and mapping of 0.67 nm/σ, we prepared two model PGN systems with relatively large particle sizes to match the size, length, and grafting density of two experimental samples.

Boundary Conditions and system size

To represent a monolayer of PGNs sitting on a substrate, while we use periodic boundary conditions for x and y, whereas periodic boundary conditions are not used for z. We use shrink wrapped boundary conditions for +z to represent the free surface, and a fixed boundary with

a wall-potential for -z. “Shrink-wrapped” is meant to invoke the fact that the box adjusts to the size of the system, but there is nothing actually confining the system in that dimension. As noted in the manuscript, interactions with the wall are handled via a 9-3 LJ potential of the form

$$U(z) = \epsilon \left[\frac{2}{15} \left(\frac{\sigma}{z} \right)^9 - \left(\frac{\sigma}{z} \right)^3 - \frac{2}{15} \left(\frac{\sigma}{z_c} \right)^9 + \left(\frac{\sigma}{z_c} \right)^3 \right], \tag{1}$$

cut and shifted to 0 at a distance of $z_c = 5.5\sigma$ (*i.e.* the energy is zero 5.5 bead diameters from the wall), which approximates the interaction of a single monomer with a perfectly smooth wall composed of a continuum of 12-6 LJ monomers of size σ. z is the distance between a bead and the wall, $\sigma = 1$, and $\epsilon = 3.5 k_B T$ for both monomer-wall and nanoparticle-wall interactions. Note that the nanoparticle-wall interactions are shifted by the same factor of 14.5σ used in the nanoparticle-monomer 12-6 LJ interactions, to roughly account for the nanoparticle size. Using the interaction strength of $3.5 k_B T$ referenced earlier the simulation cells produced have the dimensions listed below:

AuPS27: $L_x = 66.1\sigma$ (44.3 nm), $L_y = 114.4\sigma$ (76.7 nm), $L_z = 70.4\sigma$ (47.2 nm)

AuPS53: $L_x = 65.4\sigma$ (43.8 nm), $L_y = 113.2\sigma$ (75.9 nm), $L_z = 71.9\sigma$ (48.2 nm).

References

(1) Mukherjee, S.; Streit, J. K.; Gann, E.; Saurabh, K.; Sunday, D. F.; Krishnamurthy, A.; Ganapathysubramanian, B.; Richter, L. J.; Vaia, R. A.; DeLongchamp, D. M. Polarized X-ray scattering measures molecular orientation in polymer-grafted nanoparticles. *Nat Commun* **2021**, *12*, 4896.

(2) Ethier, J. G.; Hall, L. M. Modeling individual and pairs of adsorbed polymer-grafted nanoparticles: structure and entanglements. *Soft Matter* **2018**, *14*, 643–652.

(3) Ethier, J. G.; Hall, L. M. Structure and entanglement network of model polymer-grafted nanoparticle monolayers. *Macromolecules* **2018**, *51*, 9878–9889.

(4) Kröger, M. Shortest multiple disconnected path for the analysis of entanglements in two- and three-dimensional polymeric systems. *Computer physics communications* **2005**, *168*, 209–232.

(5) Karayiannis, N. C.; Kröger, M. Combined molecular algorithms for the generation, equilibration and topological analysis of entangled polymers: Methodology and performance. *International journal of molecular sciences* **2009**, *10*, 5054–5089.

(6) Hoy, R. S.; Foteinopoulou, K.; Kröger, M. Topological analysis of polymeric melts: Chain-length effects and fast-converging estimators for entanglement length. *Physical Review E* **2009**, *80*, 031803.

(7) Fetters, L.; Lohse, D.; Richter, D.; Witten, T.; Zirkel, A. Connection between polymer molecular weight, density, chain dimensions, and melt viscoelastic properties. *Macromolecules* **1994**, *27*, 4639–4647.
